# Using Polygenic Risk Scores for Prioritizing Individuals at Greatest Need of a Cardiovascular Disease Risk Assessment

**DOI:** 10.1161/JAHA.122.029296

**Published:** 2023-07-25

**Authors:** Ryan Chung, Zhe Xu, Matthew Arnold, Samantha Ip, Hannah Harrison, Jessica Barrett, Lisa Pennells, Lois G. Kim, Emanuele Di Angelantonio, Ellie Paige, Scott C. Ritchie, Michael Inouye, Juliet A. Usher‐Smith, Angela M. Wood

**Affiliations:** ^1^ British Heart Foundation Cardiovascular Epidemiology Unit, Department of Public Health and Primary Care University of Cambridge United Kingdom; ^2^ Heart and Lung Research Institute University of Cambridge United Kingdom; ^3^ Centre for Cancer Genetic Epidemiology, Department of Public Health and Primary Care University of Cambridge United Kingdom; ^4^ Medical Research Council Biostatistics Unit University of Cambridge United Kingdom; ^5^ National Institute for Health and Care Research Blood and Transplant Research Unit in Donor Health and Behaviour University of Cambridge United Kingdom; ^6^ British Heart Foundation Centre of Research Excellence University of Cambridge United Kingdom; ^7^ Health Data Research UK Cambridge Wellcome Genome Campus and University of Cambridge United Kingdom; ^8^ Health Data Science Research Centre Human Technopole Milan Italy; ^9^ National Centre for Epidemiology and Population Health Australian National University Canberra Australia; ^10^ The George Institute for Global Health UNSW Sydney Australia; ^11^ Cambridge Baker Systems Genomics Initiative, Department of Public Health and Primary Care University of Cambridge United Kingdom; ^12^ Cambridge Baker Systems Genomics Initiative Baker Heart and Diabetes Institute Melbourne Victoria Australia; ^13^ Primary Care Unit, Department of Public Health and Primary Care University of Cambridge United Kingdom; ^14^ Cambridge Centre of Artificial Intelligence in Medicine University of Cambridge United Kingdom

**Keywords:** cardiovascular disease, electronic health records, genomics, primary care records, screening, Cardiovascular Disease, Epidemiology, Primary Prevention, Risk Factors

## Abstract

**Background:**

The aim of this study was to provide quantitative evidence of the use of polygenic risk scores for systematically identifying individuals for invitation for full formal cardiovascular disease (CVD) risk assessment.

**Methods and Results:**

A total of 108 685 participants aged 40 to 69 years, with measured biomarkers, linked primary care records, and genetic data in UK Biobank were used for model derivation and population health modeling. Prioritization tools using age, polygenic risk scores for coronary artery disease and stroke, and conventional risk factors for CVD available within longitudinal primary care records were derived using sex‐specific Cox models. We modeled the implications of initiating guideline‐recommended statin therapy after prioritizing individuals for invitation to a formal CVD risk assessment. If primary care records were used to prioritize individuals for formal risk assessment using age‐ and sex‐specific thresholds corresponding to 5% false‐negative rates, then the numbers of men and women needed to be screened to prevent 1 CVD event are 149 and 280, respectively. In contrast, adding polygenic risk scores to both prioritization and formal assessments, and selecting thresholds to capture the same number of events, resulted in a number needed to screen of 116 for men and 180 for women.

**Conclusions:**

Using both polygenic risk scores and primary care records to prioritize individuals at highest risk of a CVD event for a formal CVD risk assessment can efficiently prioritize those who need interventions the most than using primary care records alone. This could lead to better allocation of resources by reducing the number of risk assessments in primary care while still preventing the same number of CVD events.

**Nonstandard Abbreviations and Acronyms**
CPRD: Clinical Practice Research Datalink
NNS: number needed to screen
PRS: polygenic risk score
UKB: UK Biobank

**Clinical Perspective**

**What Is New?**

- Data from a large prospective cohort study were used to assess the public health impact of using polygenic risk scores for systematically identifying individuals for invitation for full formal cardiovascular disease risk assessment.
- Our study directly compared the use of tools derived using primary care records or polygenic risk scores in the same population.
- Our study quantified a 20% to 35% reduction in the number needed to screen to prevent 1 CVD event by using polygenic risk scores at both the invitation and full formal CVD assessment stage.

**What Are the Clinical Implications?**

- These results provide quantitative evidence of current guidelines in England to systematically prioritize individuals using existing primary care records.
- Using polygenic risk scores may be beneficial in identifying high‐risk individuals before a full formal risk assessment, which could lead to better allocation of resources by reducing the number of formal risk assessments in primary care while still preventing the same number of cardiovascular disease events.

Cardiovascular disease (CVD) remains a major cause of morbidity and death worldwide.^1^ Identifying individuals at a high risk of CVD to manage and implement interventions to reduce risk of CVD remains an important aim.^2^, ^3^ Prediction tools using the risk factor levels of individuals to estimate a 5‐ or 10‐year risk of CVD have been developed to aid clinical decision‐making and are recommended by health care guidelines across the world.^3^, ^4^, ^5^, ^6^, ^7^, ^8^, ^9^, ^10^ However, recent studies have debated the clinical value and cost effectiveness of national risk assessment programs.^11^, ^12^, ^13^, ^14^, ^15^, ^16^, ^17^ In line with this, recent guidelines have made recommendations to better use existing primary care records to improve the stratification of high‐risk individuals before formal CVD risk assessments.^18^ However, few strategies or tools to systematically identify such individuals have been recommended. Proposals have also been recommended to prioritize individuals using CVD‐based polygenic risk scores (PRSs); such PRSs have been shown to be independent of other CVD risk factors, offering improved stratification with high concordance between categories of polygenic risk and future CVD risk across the life course, and to improve discriminatory performance when used to supplement existing CVD risk scores.^19^, ^20^, ^21^ However, no studies have quantified the impact PRSs would have for prioritization.

Therefore, to investigate the benefits of PRSs to systematically prioritize individuals at high risk of CVD, we compare systematically prioritizing individuals using a PRS‐based prioritization tool against current guideline recommendations of using a prioritization tool that is based on longitudinal primary care records.^22^, ^23^, ^24^, ^25^, ^26^

## Methods

### Ethics Approval

This research has been conducted using the UK Biobank Resource under Application Number 26865. Data from the Clinical Practice Research Datalink (CPRD) were obtained under license from the UK Medicines and Healthcare Products Regulatory Agency (protocol 162RMn2).

### Data Availability

All data files are available from the UK Biobank and CPRD databases.

### UK Biobank Data Source

UKB (UK Biobank) is a prospective cohort study with detailed baseline information, genetic data, and linked primary care record data available for 177 359 individuals in England recruited between 2006 and 2010.^27^ Genetic data were sequenced using a genome‐wide array of ≈826 000 markers with imputation to ≈96 million markers.^27^ Primary care data were provided by the Phoenix Partnership, Egton Medical Information Systems, and Vision GP system suppliers.^28^ Data were linked with secondary care admissions from Hospital Episode Statistics and death records from the Office for National Statistics. For this study, primary care records were restricted to those measured between April 1, 2004, and the introduction of the Quality and Outcomes Framework and UKB baseline survey. To assess the impact of PRSs as a prioritization tool and compare with primary care records, our primary analyses were restricted to individuals with complete genetic data necessary for calculating the PRS, at least 1 primary care record, and without prior CVD or statin initiation before UKB baseline. Individuals contributing to the PRS derivation were also excluded. Data from the UKB were used to derive CVD risk tools and to model the implications of prioritizing individuals for formal assessment (Figure S1). All individuals gave informed consent.

### CPRD Data Source

The Clinical Practice Research Datalink (CPRD) is a large UK primary care database containing primary care records^28^ with linked information from Hospital Episode Statistics and death records from the Office for National Statistics. The most recent 5‐year primary care records available were extracted for 870 486 individuals who were still alive and without prior CVD on January 1, 2014, and had no statins throughout follow‐up until May 31, 2019, the end of data availability (Figure S2). Data from CPRD were used to rescale estimated CVD risks in UKB participants to address the healthy cohort effect (Figure S1). All individuals gave informed consent.

### Outcomes

CVD was defined as the first ever incident of fatal or nonfatal events of coronary heart disease (including angina and myocardial infarction), ischemic heart disease, and stroke (code lists provided in Table S1), appearing in the linked Hospital Episode Statistics and Office for National Statistics databases during follow‐up.

### Risk Factors

Two PRSs for coronary artery disease and stroke, constructed using a meta‐score approach and external summary statistics from large genome‐wide association studies,^20^, ^29^ were used as independent variables. Conventional risk factors (as those in the QRISK2 scores^4^) were selected: age; sex; ethnicity; Townsend score; smoking status (current/ever smoker); history of diabetes (type 1 or type 2 or history of diabetes medication); family history of CVD; history of chronic kidney disease (stages 4 and 5); history of atrial fibrillation status; history of blood pressure treatment; history of rheumatoid arthritis; total and high‐density lipoprotein (HDL) cholesterol; systolic blood pressure (SBP); body mass index (BMI); and age interactions with Townsend score, history of diabetes, family history of CVD, history of atrial fibrillation, history of blood pressure treatment, SBP, and BMI.

### Statistical Modeling

Sex‐specific Cox models were used to derive 3 different prioritization tools for estimating 10‐year CVD prioritization risk using primary care and genetic data from UKB. First, we derived a prioritization tool with linear predictors of baseline age, coronary artery disease PRS,^20^ and stroke PRS.^29^ Age interactions were considered but were not statistically significant at the 5% level. Second, we derived a prioritization tool with predictors using longitudinal primary care records. To handle missing values, the tool was derived in 2 stages: In the first stage, we used sex‐specific multivariate mixed‐effect regression models on longitudinal risk factor measurements for SBP, total and HDL cholesterol, and BMI to estimate current risk factor values (Data S1); in the second stage, we derived sex‐specific Cox models with the estimated current risk factor values for SBP, total and HDL cholesterol, and BMI, and the most recent primary care measurements for the remaining QRISK2 risk factors. Third, we derived a prioritization tool with both PRSs and primary care records, using the 2‐stage approach described above with the addition of linear predictors for the coronary artery disease PRS and stroke PRS in the second‐stage Cox models. For each of these 3 tools, the model is used to identify individuals crossing a minimum 10‐year risk threshold to be invited for a formal assessment.

Sex‐specific Cox models were used to derive 2 formal risk assessment models for predicting 10‐year formal assessment CVD risk using risk factor measurements recorded at UKB baseline survey. First, we rederived a model based on QRISK2 predictors; and second, we derived a model based on QRISK2 predictors enhanced with the coronary artery disease PRS and stroke PRS.

All models were validated using 10‐fold cross validation, and prognostic ability was quantified using Harrell's C‐index to measure discrimination.

### Population Health Modeling

**Figure 1:**
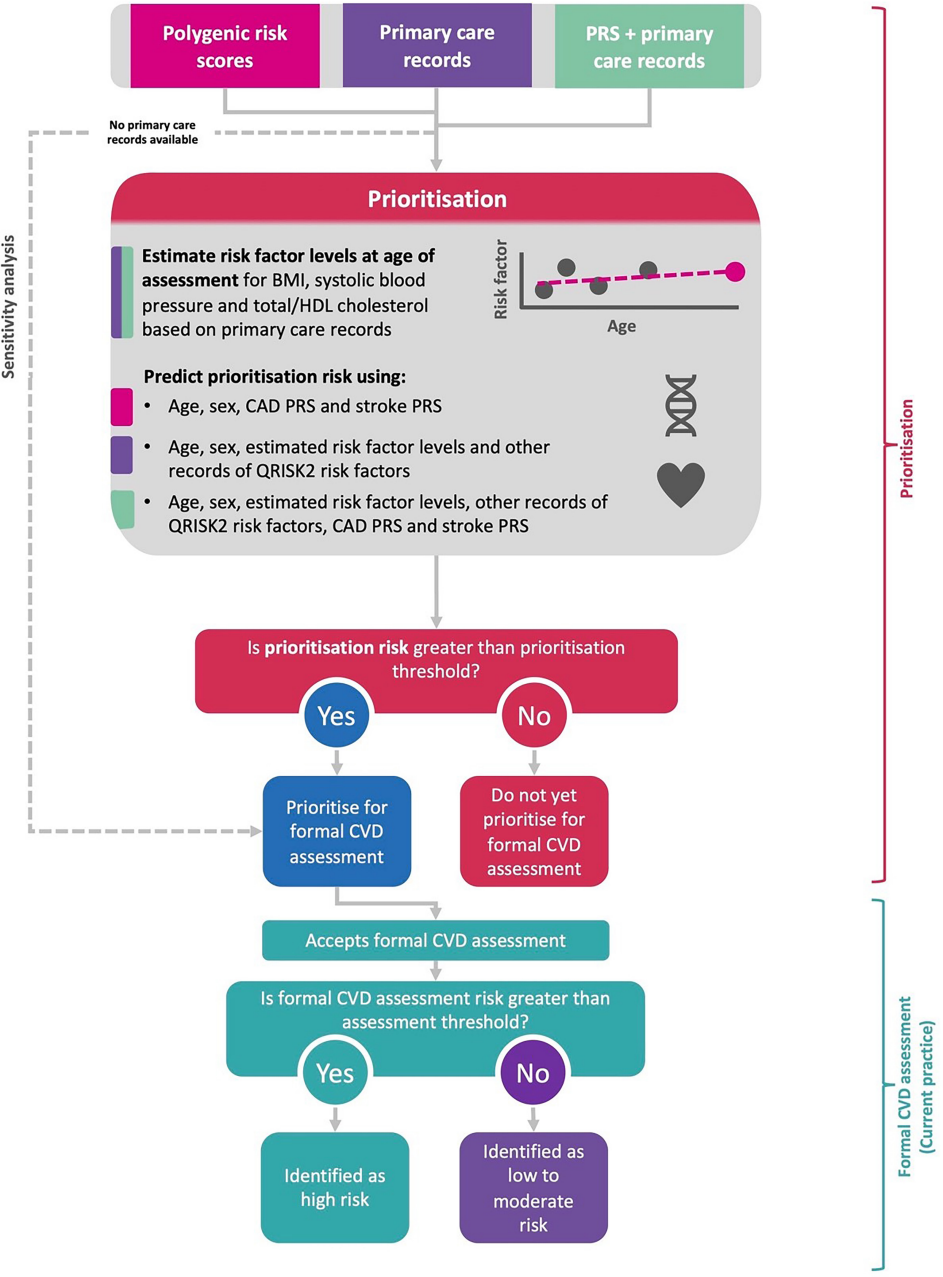
Flowchart of the implementation of a prioritization tool for formal cardiovascular disease assessments. BMI indicates body mass index; CAD, coronary artery disease; CVD; cardiovascular disease; HDL, high‐density lipoprotein; and PRS, polygenic risk score.

Population health modeling was conducted to compare the population health impact of (1) prioritizing using a primary care records–based tool followed by a formal assessment with conventional risk factors, (2) prioritizing using a PRS and age‐based tool followed by a formal assessment with conventional risk factors and PRS, and (3) prioritizing using both PRS and primary care records, followed by a formal assessment with conventional risk factors and PRSs (Figure 1). As UKB consists of healthier individuals than the general primary care population in England, we rescaled each model's estimated CVD risks so that the distribution of risks estimated were using age group– and sex‐ specific level risk factors obtained from CPRD and the published QRISK2 score to better reflect the CVD risk assessment program in the general population (Data S2; Table S2 and Table S3). Details of the rescaling method have been described elsewhere.^30^, ^31^

A hypothetical population of 100 000 individuals (50 000 men and 50 000 women) from the United Kingdom was created; the population age structure was obtained using data from the Office for National Statistics in 2015,^32^ and the number of expected CVD events was calculated using age group– and sex‐specific incidence rates from CPRD (Table S3; Figure S3). A policy of statin initiation for individuals at ≥10% predicted 10‐year formal assessment of CVD risk as currently recommended by National Institute for Health and Care Excellence guidelines and a 20% reduction in CVD risk were assumed.^33^, ^34^ The population health impact for each of the 3 prioritization tools was modeled using age‐ and sex‐specific prioritization thresholds in 2 ways. First, we selected prioritization thresholds to limit the formal risk assessment false‐negative rate to 5%. Second, we selected prioritization thresholds for the tools using PRSs, such that the same number of events identified would be equivalent to prioritizing with primary care records (Table S4).

Summary metrics were estimated for the number needed to screen (NNS) to prevent 1 CVD event, the number of CVD events identified, and the number needed to invite to prevent 1 CVD event. We assumed 50% statin compliance and a 50% invitation uptake of a formal assessment if inviting all individuals.^35^, ^36^ We further assumed an increased invitation uptake of 55% if individuals were prioritized for an invitation to a formal assessment. Bootstrap 95% CIs were calculated using 500 iterations.

In sensitivity analyses, we repeated population‐health analyses for all individuals, including those without a primary care record for any 1 of SBP, HDL, total cholesterol, or BMI, where those without a record were all invited for formal assessment (Table S3). We also repeated analyses assuming a 5% formal risk assessment threshold, in addition to age‐ and sex‐specific prioritization thresholds selected to correspond to 2.5% false‐negative rates.

Analyses were conducted in R x64 3.6.1 (R Foundation for Statistical Computing, Vienna, Austria). This study follows the RECORD statement (Table S5).^37^

## Results

### Population Characteristics

For our primary analysis, we identified 108 685 individuals in UKB with genetic data and a primary care record for at least 1 of SBP, HDL, total cholesterol, and BMI (Figure S4). All individuals had complete information for the conventional risk factors necessary to calculate a 10‐year formal CVD risk at baseline survey.

The mean age at baseline was 56.2 years (SD, 8.0) for men and 56.1 years (SD, 7.8) for women. During mean follow‐up of 8.2 years, there were 1838 incident cardiovascular events (Table 1). Compared with the measurements observed at the UKB baseline survey, the measurements recorded in primary care records were lower for SBP and total cholesterol and, although similar for current/ever smoking status and history of diabetes, were less concordant for the remaining disease statuses. The oldest primary care record was on average 3.8 years before baseline.

**Table 1:** Key Characteristics of Individuals in UK Biobank Baseline Survey and Linked Primary Care Records

Characteristic	Men, N=44 184 (41%)	Women, N=64 501 (59%)
CVD events, N	1230	608
Follow‐up duration, y, median (5th–95th percentile)	8.1 (6.1–10.8)	8.2 (6.8–10.9)
Duration between first primary care record and baseline visit, y, median (5th–95th percentile)	3.6 (0.9–5.5)	3.9 (1.1–5.6)

	Primary care records	Baseline	Primary care records	Baseline
Age, y, mean (SD)*	…	56.2 (8.0)	…	56.1 (7.8)
Coronary artery disease PRS, mean (SD)	…	−1.15 (0.46)	…	−1.13 (0.46)
Stroke PRS, mean (SD)	…	1.55 (0.22)	…	1.56 (0.23)
Ethnicity, White, N (%)*	…	42 283 (95.7)	…	61 977 (96.1)
Townsend, mean (SD)*	…	−1.5 (3.0)	…	−1.5 (2.9)
Systolic blood pressure, mm Hg, mean (SD)†	135.3 (7.82)	141.0 (17.3)	130.7 (9.62)	134.9 (19.1)
Number of historical records, mean	3.8	…	4.6	…
Total cholesterol, mmol/L, mean (SD)†	5.48 (0.47)	5.79 (1.01)	5.71 (0.50)	6.03 (1.08)
Number of historical records, mean	2.0	…	2.0	…
HDL cholesterol, mmol/L, mean (SD)†	1.35 (0.19)	1.30 (0.31)	1.68 (0.24)	1.61 (0.37)
Number of historical records, mean	1.8	…	1.9	…
BMI, kg/m^2^, mean (SD)†	27.2 (3.1)	27.5 (4.1)	26.6 (4.1)	26.8 (5.0)
Number of historical records, mean	2.0	…	2.3	…
Current/ever smoker, N (%)	4472 (10.1)	5233 (11.8)	4911 (7.61)	5511 (8.5)
History of diabetes, N (%)	466 (1.05)	630 (1.4)	412 (0.64)	459 (0.7)
Blood pressure–lowering medication prescriptions, N (%)	6396 (14.5)	5529 (12.51)	9737 (15.1)	7643 (11.85)
Family history, N (%)*	1568 (3.55)	…	2494 (3.87)	…
Chronic kidney disease (4/5), N (%)*	57 (0.13)	…	79 (0.12)	…
Rheumatoid arthritis, N (%)	146 (0.33)	381 (0.86)	336 (0.52)	989 (1.53)
Atrial fibrillation, N (%)	336 (0.33)	123 (0.28)	1749 (2.7)	89 (0.14)

BMI indicates body mass index; CVD, cardiovascular disease; HDL, high‐density lipoprotein; and PRS, polygenic risk score.

* Risk factor values in both baseline and primary care records if 1 was missing.

^†^ Risk factor values for primary care records estimated using multivariate mixed‐effect model.

### Model Performance and Comparison

Hazard ratios in the prioritization tools and formal assessment models for the same predictors were similar (Tables S6 and S7). All models had good discriminatory performance, with higher performance in women (Table 2). The greatest performance was observed in the model using conventional risk factors and PRSs in men (C‐index, 0.716; 95% CI, 0.702–0.730) and in women (C‐index, 0.742; 95% CI, 0.722–0.762).

**Table 2:** C Indices of Prioritization Tools and Formal CVD Risk Assessment Tools in UK Biobank

Model	C‐index (95% CI)		
	All individuals	Men	Women
Prioritization tool			
Primary care records only	0.730 (0.719–0.741)	0.684 (0.670–0.699)	0.734 (0.715–0.754)
PRS+age	0.663 (0.652–0.675)	0.663 (0.649–0.678)	0.686 (0.665–0.707)
PRS+primary care records	0.740 (0.730–0.751)	0.704 (0.691–0.718)	0.738 (0.718–0.758)
Formal risk assessment tool			
Conventional risk factors	0.730 (0.719–0.740)	0.700 (0.686–0.714)	0.739 (0.720–0.759)
Conventional risk factors+PRS	0.738 (0.727–0.749)	0.716 (0.702–0.730)	0.742 (0.722–0.762)

C‐indices and 95% CIs from each model for the prediction of 10‐year cardiovascular disease by sex and for the combined population in UK Biobank after 10‐fold cross validation. CVD indicates cardiovascular disease; and PRS, polygenic risk score.

The estimated 10‐year risks between the primary care records–only prioritization tool and the formal assessment model using conventional risk factors were highly correlated (correlation coefficients, 0.75 for men and 0.80 for women). In contrast, the estimated 10‐year risks between the PRS+age prioritization tool and the formal assessment model using conventional risk factors and PRS were less highly correlated (correlation coefficients, 0.67 for men and women), and the estimated 10‐year risks between the PRS and primary care records based prioritization tool and the formal assessment model using conventional risk factors with PRS were more highly correlated (correlation coefficients, 0.82 for men and women; Table 3). Rescaled 10‐year risk estimates between all models were similar (Figure S5).

**Table 3:** Correlation of Predicted 10‐Year Risks Between Prioritization Tools and Formal Assessment Tools by Sex in the Derivation Data Set

	Primary care records–only prioritization tool	PRS+age prioritization tool	PRS+primary care records prioritization tool
Men			
Conventional risk factor formal assessment tool	0.75	…	…
Conventional risk factor+PRS formal assessment tool	…	0.67	0.82
Women			
Conventional risk factor formal assessment tool	0.80	…	…
Conventional risk factor+PRS formal assessment tool	…	0.67	0.82

PRS indicates polygenic risk score.

### Population Health Modeling

**Figure 2:**
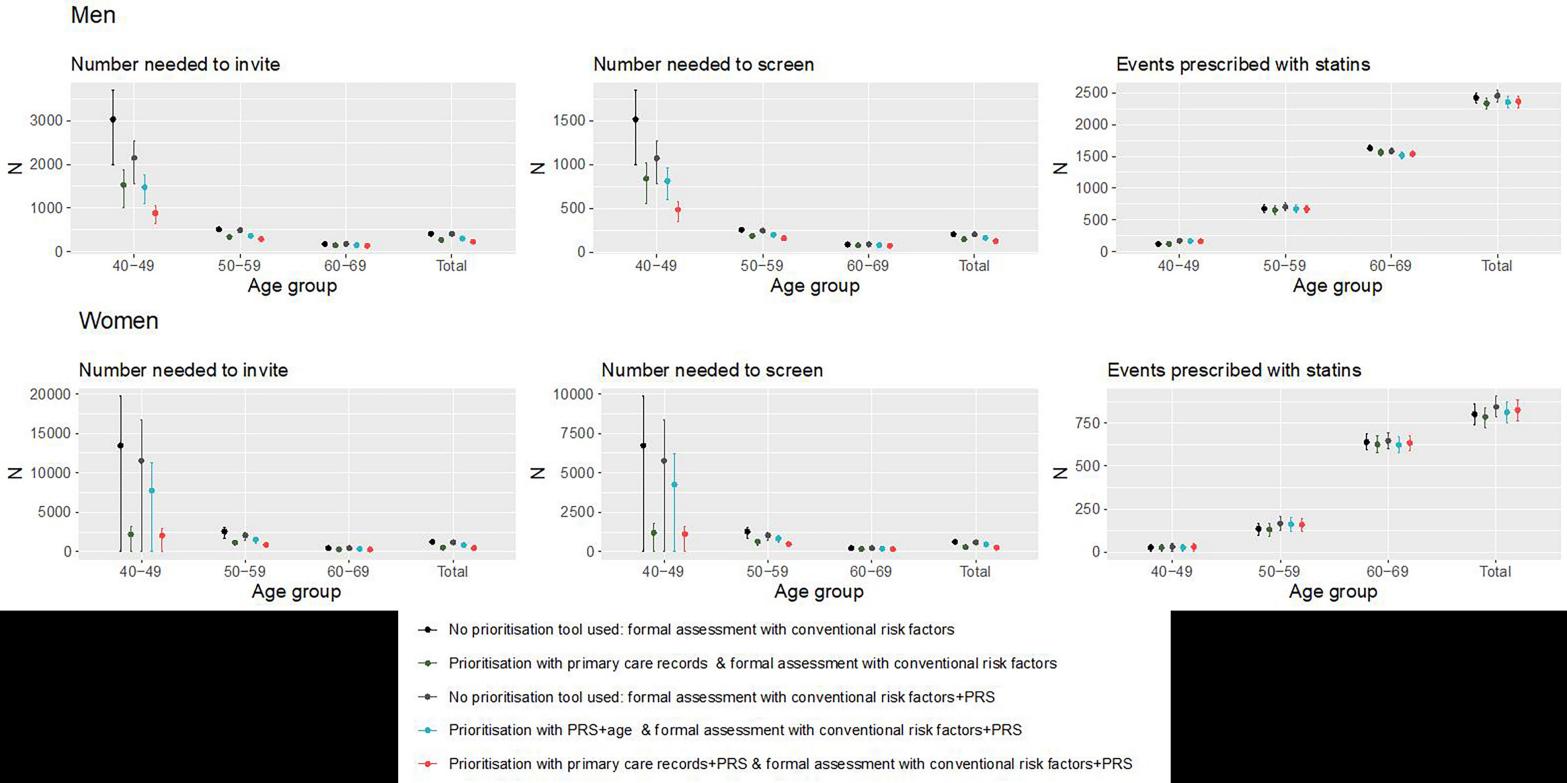
Number needed to invite, number needed to screen, and number of events identified after prioritizing for a formal CVD assessment, in a hypothetical population of 100 000 individuals in England. 95% CIs are represented by vertical lines. Age group– and sex‐specific prioritization thresholds were defined as the level such that the expected false‐negative rate is controlled to be 5%. NNI and NNS assumes 50% statin compliance, and half of all individuals invited for formal assessment attend. CVD indicates cardiovascular disease; NNI, number needed to invite; NNS, number needed to screen; and PRS, polygenic risk score.

In our representative population of 100 000 individuals aged 40 to 69, 3573 men and 1808 women would experience a CVD event over the next 10 years. If conventional risk factors were used to formally assess the whole population, 2426 (67.9%) men and 801 (44.3%) women would be identified at high risk (Figure 2, Table S8). Assuming statin therapy would be initiated on high‐risk individuals and no other preventive interventions implemented, the NNS to prevent 1 CVD event in men and women would be 103 (95% CI, 100–107) and 312 (95% CI, 288–334), respectively. If the primary care records–based prioritization tool was first used to prioritize formal assessment in the population, then 2335 (65.3%) men and 785 (43.4%) women would be identified at high risk (Figure 2, Table S8). The NNS to prevent 1 event would reduce to 149 (95% CI, 143–155) in men and 280 (95% CI, 259–301) in women (27.7% and 55.1% reduction, respectively).

If conventional risk factors enhanced with PRS was used to formally assess the whole population, then 2457 (68.8%) men and 844 (46.7%) women would be identified as being at high risk (Figure 2, Table S9). The NNS to prevent 1 CVD event in men and women would be 204 (95% CI, 197–211) and 592 (95% CI, 545–631), respectively.

If the PRS+age prioritization tool was first used to prioritize formal assessment in the population, then 78.8% of men and 74.8% of women would be prioritized and, among them, 2356 (65.9%) men and 813 (45.0%) women with CVD events over the next 10 years would be classified at high risk (Figure 2, Table S9). The NNS to prevent 1 event would reduce to 167 (95% CI, 161–174) in men and 460 (95% CI, 423–491) in women (18.1% and 22.3% reduction, respectively). If the PRS and primary care records–based prioritization tool was first used to prioritize formal assessment in the population, then 2367 (66.3%) men and 825 (45.6%) women would be classified at high risk (Figure 2, Table S10). The NNS to prevent 1 event would reduce to 127 (95% CI, 122–132) in men and 255 (95% CI, 234–273) in women (37.7% and 56.9% reduction, respectively).

Choosing prioritization thresholds such that all strategies would identify the same number of events if prioritizing using primary care records (Table S4) or prioritizing using PRS and age resulted in an NNS of 164 (95% CI, 157–170) in men and 446 (95% CI, 410–480) in women. Prioritizing using PRSs and primary care records resulted in an NNS of 116 (95% CI, 111–121) in men and 180 (95% CI, 166–193) in women (Table 4, Figure S6). Compared with using primary care records, prioritizing using PRSs and primary care records led to statistically significant differences in the NNS at the 5% level for all except in women aged 40 to 49 years.

**Table 4:** Number Needed to Invite and Screen to Prevent 1 Event and Number of Events Identified After Prioritization and Formal Assessment in a Hypothetical Population of 100 000 Individuals in England, With Prioritization Thresholds Selected to Identify the Same Number of Events if Prioritizing With Primary Care Records With Prioritization Thresholds Controlling the False Negative Rate to 5%

		Prioritization using primary care records followed by conventional risk factors				Prioritization using PRS+age, followed by conventional risk factors+PRS				Prioritization using PRS and primary care records, followed by conventional risk factors+PRS			
Age group	Participants	Participants prioritized, n (%)	NNI (95% CI)	NNS (95% CI)	Number of events identified as high risk, n (%)	Participants prioritized, n (%)	NNI (95% CI)	NNS (95% CI)	Number of events identified as high risk, n (%)	Participants prioritized, n (%)	NNI (95% CI)	NNS (95% CI)	Number of events identified as high risk, n (%)
Men													
40–49	18 253	10 126 (55.5)	1530 (997.9–1865.6)	841 (548.9–1026.1)	120 (24.8)	13 016 (71.3)	1454 (1060.7–1713.0)	800 (583.4–942.2)	163 (33.6)	7208 (39.5)	738 (531.5–876.8)	406 (292.3–482.2)	163 (33.6)
50–59	17 391	12 134 (69.8)	339 (301.2–368.3)	187 (165.7–202.6)	651 (52.5)	13 100 (75.3)	358 (325.1–386.6)	196 (178.8–212.6)	666 (53.7)	9878 (56.8)	266 (236.7–288.8)	146 (130.2–158.9)	654 (52.7)
60–69	14 356	12 517 (87.2)	146 (140.8–149.8)	80 (77.5–82.4)	1564 (84.7)	12 196 (84.9)	148 (141.7–152.2)	80 (77.9–83.7)	1506 (81.5)	11 064 (77.1)	130 (124.2–133.7)	72 (68.3–73.6)	1519 (82.3)
Total	50 000	34 777 (69.6)	271 (260.7–281.1)	149 (143.4–154.6)	2335 (65.3)	38 313 (76.6)	298 (286.0–309.9)	164 (157.3–170.4)	2335 (65.4)	28 150 (56.3)	210 (200.9–219.1)	116 (110.5–120.5)	2336 (65.4)
Women													
40–49	18 107	3233 (17.9)	2185 (0.0–3192.3)	1202 (0.0–1755.7)	27 (10.0)	10 139 (56.0)	7100 (0.0–10397.2)	3904 (0.0–5718.5)	27 (10.0)	1748 (9.7)	1012 (0.0–1462.2)	558 (0.0–804.2)	31 (11.7)
50–59	17 282	8329 (48.2)	1143 (778.9–1403.4)	629 (428.4–771.9)	132 (23.0)	12 436 (72.0)	1484 (1013.9–1783.4)	816 (557.7–980.9)	156 (27.1)	4683 (27.1)	612 (397.5–746.1)	336 (218.6–410.3)	139 (24.1)
60–69	14 611	10 459 (71.6)	304 (277.4–324.1)	167 (152.6–178.3)	626 (65.1)	11 357 (77.7)	356 (326.4–379.5)	196 (179.5–208.7)	603 (62.7)	7714 (52.8)	228 (210.4–242.5)	126 (115.7–133.4)	616 (64.0)
Total	50 000	22 021 (44.0)	510 (470.8–547.0)	280 (258.9–300.8)	785 (43.4)	33 932 (67.9)	812 (744.7–872.1)	446 (409.6–479.6)	786 (43.5)	14 145 (28.3)	326 (301.0–351.0)	180 (165.6–193.0)	786 (43.5)

Age structure of hypothetical population extrapolated from Office for National Statistics, England, United Kingdom, 2015. Expected events at 10 years based on extrapolation of incidence rates from CPRD, 2014–2019. Age group– and sex‐specific prioritization thresholds when prioritizing using primary care records were defined as the level such that the expected false‐negative rate is controlled to be 5%. Age group– and sex‐specific prioritization thresholds when prioritizing using PRS or PRS and primary care records tool were selected to result in the same number of events identified if prioritizing using primary care records. NNI and NNS assumes 100% statin compliance. NNI assumes a 50% invitation uptake if assessing without using prioritization tool, and a 55% invitation uptake if assessing with using prioritization tool. Bootstrap 95% CIs were calculated using 500 iterations. NNI indicates number needed to invite; NNS, number needed to screen; and PRS, polygenic risk score.

### Sensitivity Analysis

In sensitivity analyses including all individuals (ie, including 15 324 individuals without a primary care record for any 1 of SBP, total cholesterol, HDL cholesterol, or BMI; Table S11), we found comparable results for the PRS‐based prioritization tool and the primary care–based prioritization tool in men and women. As expected, we increased the NNS if prioritizing with primary care records, especially among the youngest group (Tables S12 through S14, Figure S7).

In sensitivity analyses assuming a 5% formal risk assessment threshold, in addition to age‐ and sex‐specific prioritization thresholds selected to correspond to 2.5% false‐negative rates (Table S15), we found significant improvements in the number of events identified, as expected. Consequently, this reduces the NNS when formally assessing all individuals, using either conventional risk factors or conventional risk factors enhanced with PRSs (Tables S16 through S18). While prioritization can still reduce the overall NNS and the NNS among the youngest, the differences are smaller compared with when using a 10% formal risk assessment threshold.

## Discussion

This study has rigorously assessed the impact of using PRS both alone and in combination with traditional risk factors for systematically prioritizing individuals for a formal CVD risk assessment. Comparing against current recommendations of using existing primary care records, we found that adding PRS to both prioritization and formal assessment improves their correlation. This subsequently leads to higher efficiency and effectiveness, especially among younger individuals. Consequently, augmenting primary care records with PRSs reduces the NNS by ≈20% and 35% in men and women, respectively, relative to using primary care records alone and identifying the same number of events. In contrast, using only PRS and age in a prioritization tool leads to a larger NNS. These results support the addition of PRS with primary care records to prioritize individuals at highest risk for a formal CVD risk assessment, which could lead to better allocation of resources by reducing the number of assessments.

This study provided a comparison of prioritization tools using longitudinal primary care records or PRSs within a population in England aged between 40 and 69 years who are currently invited for a National Health Service Health Check to assess their individual risk of CVD. We demonstrated the benefits of PRS not only by measuring model discrimination but also by evaluating the health impact if implemented within this population. Compared with previous studies that generally focused on the role of PRSs in a formal CVD risk assessment model,^20^, ^21^, ^29^, ^38^ our study has uniquely assessed its role in a prioritization tool, in conjunction with a CVD risk model. We have also shown that if PRSs were widely available, the inclusion of PRSs in a prioritization tool could improve the effectiveness of a prioritization tool, especially in younger individuals, by reducing the reliance on primary care records.

The benefits in prioritizing a subgroup of those individuals at low absolute risk to increase efficiency echoes other studies, which have also shown that selecting a smaller proportion of younger, low‐risk individuals can lead to dramatically reduced costs while resulting in more quality‐adjusted life years gained.^39^ Using a prioritization tool could also efficiently help reduce the concerning backlog in health checks caused by the COVID‐19 pandemic,^40^ where the number of people invited to health checks in England declined by 82% between the end of 2019 and 2020,^41^, ^42^, ^43^ while still preventing nearly the same number of CVD events. While the addition of PRSs has the potential to prioritize individuals earlier for a formal CVD assessment, further extensions include using PRSs to identify individuals at high risk of other common chronic diseases, including diabetes, dementia, and kidney disease.^44^, ^45^, ^46^, ^47^, ^48^

### Strengths and Limitations

Our study has several strengths. To our knowledge, this study is the first to directly compare how using different data types for a prioritization tool can impact on the CVD risk assessment program in England. This was possible due to the unique data linkage of primary care records along with a baseline survey in UKB. We derived the PRS‐based prioritization tools using 2 current and well‐documented PRSs that have been shown to improve model performance independent of traditional CVD risk factors. We also took advantage of the sporadically observed longitudinal primary care records when deriving the primary care records–based prioritization tools, by estimating current risk factor values using a multivariate mixed model. While QRISK2, which replaces missing values with age‐, sex‐, and ethnicity‐specific population average values, could have been used as a prioritization tool, we chose to optimize the available data in primary care records to reduce possible overinflation of the information from PRSs. We would expect greater improvements if augmenting PRSs in CVD risk scores with fewer risk factors. Another strength of this study is the use of 10‐fold cross validation to correct for overoptimism that may exist in our analyses, as we derived and conducted the population health modeling in the same individuals. Further, we used rescaling methods to adjust the 10‐year risk estimates for all of the models to minimize the healthy selection bias when deriving models in UKB and to ensure that results were representative to the general population of England. Such rescaling methods could be adapted toward other countries.

However, several potential limitations exist. First, while we used primary care records that were no more than 6.5 years old before baseline, the mean risk factor levels between primary care records and at the UKB baseline differed within the same individuals, which could lead to a different distribution of 10‐year risk estimates. This may also weaken the correlations between the prioritization tool and formal risk assessment models reported. Second, we determined the number of events identified in the population health modeling by calculating the model's sensitivity in the UKB and translating to a hypothetical population; due to the low number of events in the UKB, the sensitivity of each model may be limited in accuracy, especially in younger age groups with fewer events. In addition, any uncertainties are propagated within the population health modeling. Third, PRSs for cardiovascular disease are still under active development, and while we use 2 extensively studied and validated PRSs, there are likely more powerful PRSs soon to be available.^49^ Fourth, the age range of the population health modeling was limited to between 40 and 69 years due to the use of UK Biobank. This restricts the population health modeling and, in particular, limits the ability to investigate the early prioritization capabilities of PRSs (which are fixed at conception). Fifth, we have focused on estimating the differences in a primary care population in England. Further work should generalize the findings to other countries and their respective health care systems. Sixth, we assumed a constant 20% reduction in risk due to statins, which is unlikely in practice, where reductions may be greater in those with a greater genetic risk.^50^ Seventh, while data from CPRD are generally representative of the primary care attending population in the United Kingdom in terms of age, sex, and ethnicity, the CPRD data used do not have comprehensive coverage in the North and East of England.^28^, ^51^ Eighth, we did not model the combined effects of other preventative interventions, such as lifestyle advice. It is likely that the benefits of communicating polygenic risk may lead to beneficial lifestyle changes, which may impact health outcomes.^52^ Finally, we acknowledge our use of *International Classification of Diseases, Tenth Revision* (*ICD‐10*) codes to identify CVD outcomes may have missed some events, although this is unlikely to affect our between‐model comparisons.

## Conclusions

Population health guidelines in England recommend individuals at higher estimated risk of CVD be prioritized for formal risk assessment. Our results show that incorporating PRSs improves the correlation between prioritization tools and formal CVD risk assessment models. In particular, the use of PRSs together with primary care records to prioritize individuals at highest risk of a CVD event for a formal CVD risk assessment has the ability to efficiently prioritize those who need interventions the most, which could lead to better allocation of resources by reducing the number of formal risk assessments in primary care.

## Sources of Funding

This work was supported by core funding from the: British Heart Foundation (RG/13/13/30194; RG/18/13/33946), BHF Cambridge Centre of Research Excellence (RE/13/6/30180), and National Institute for Health and Care Research (NIHR) Cambridge Biomedical Research Centre (BRC‐1215‐20 014) [*]. *The views expressed are those of the author(s) and not necessarily those of the NIHR, National Health Service Blood and Transplant (NHSBT), or the Department of Health and Social Care. This work was funded by the Medical Research Council (MR/K014811/1). The study funders played no role in the design, analysis, or interpretation of the study. R. Chung was funded by a BHF PhD studentship (FS/18/56/34177). Dr Xu was funded by the Chinese Scholarship Council. Dr Arnold was funded by a British Heart Foundation Programme Grant (RG/18/13/33946). Dr Ip was funded by a BHF‐Turing Cardiovascular Data Science Award (BCDSA/100 005) and by a University College London FB Cancer Research UK Award (C18081/A31373). Dr Harrison was funded by an International Alliance for Cancer Early Detection Project Award (ACEDFR3_0620I135PR007). Dr Barrett was funded by a Medical Research Council fellowship (MR/L501566/1) and unit program (MC_UU_00002/5). Dr Pennells was funded by a British Heart Foundation Programme Grant (RG/18/13/33946). Dr Kim was funded by the NIHR BTRU in Donor Health and Genomics (NIHR BTRU‐2014–10 024) and by the NIHR BTRU in Donor Health and Behavior (NIHR203337) [*]. Dr Inouye was supported by the Munz Chair of Cardiovascular Prediction and Prevention and the NIHR Cambridge Biomedical Research Centre (BRC‐1215‐20 014) and EU Horizon 2020 (No 101016775 INTERVENE). Dr Inouye was also supported by the UK Economic and Social Research 878 Council (ES/T013192/1). Dr Usher‐Smith was funded by an NIHR Advanced Fellowship (NIHR300861). Dr Wood is part of the BigData@Heart Consortium, funded by the Innovative Medicines Initiative‐2 Joint Undertaking under grant agreement No 116074. Dr Wood was supported by the BHF‐Turing Cardiovascular Data Science Award (BCDSA/100 005).

## Disclosures

During the drafting of the manuscript, Dr Arnold became an employee of AstraZeneca. The remaining authors have no disclosures to report.

## Supporting information

Data S1
Tables S1–S18
Figures S1–S7
References53–55
