# Supplementary material for: Using Polygenic Risk Scores for Prioritizing Individuals at Greatest Need of a Cardiovascular Disease Risk Assessment
Source: J Am Heart Assoc. 2023 Jul 25;12(15):e029296. doi: 10.1161/JAHA.122.029296 (PMC7614905; doi:10.1161/JAHA.122.029296)
Supplement: Supplementary file 1 — Data S1 Tables S1–S18 Figures S1–S7 References53–55 [file JAH3-12-e029296-s001.pdf]

Supplemental Material

Supplemental Methods

Data S1.

Summarising repeated measures of risk factors in primary care records using multivariate mixed-effects linear regression models.

Derivation of prioritisation tools using primary care records.

Our aim was to derive a prioritisation tool to estimate a 10-year CVD risk for CVD event free individuals using their primary care records only and to then compare with a PRS-based prioritisation tool and one that combines the two together. When utilising longitudinal data in primary care records, the model needs to handle the repeated and sporadic structure of the data.

We used the same risk factors as used in the QRISK2 risk score to derive the primary care records-based prioritisation tool. Values of systolic blood pressure, total and HDL cholesterol, BMI and Townsend score were standardised using sex-specific means and standard deviations. All remaining risk factors were listed as indicator variables and were set to zero until a health record indicated otherwise.

Using the primary care records before baseline survey in a population without prior CVD or diabetes, but including those with prior statin usage, sex-specific multivariate mixed effects models with a fixed slope, random intercept and an intra-correlation structure were used to estimate the risk factor levels at the same timepoint as when the individual attended the UK Biobank baseline assessment. Let SBP_{ij}, $Total\ cholesterol_{ij}$, $HDL\ cholesterol_{ij}$, BMI_{ij} , age_{ij} , age_{ij}^2 , AHM_{ij} , and $statin_{ij}$ denote, respectively, the repeat measures of systolic blood pressure, total cholesterol, HDL cholesterol, BMI, age at visit in years, age at visit in years squared, an indicator for history of anti-hypertensive medication, and an indicator for history of statin medication for individual i and measurement j. The sex-specific multivariate mixed models and its corresponding correlated covariance structure were of the following form:

$$SBP_{ij} = a_1 + b_1 age_{ij} + c_1 age_{ij}^2 + (d * AHM_{ij}) + u_{1i} + e_{1ij}$$

$$Total\ cholesterol_{ij} = a_2 + b_2 age_{ij} + c_2 age_{ij}^2 + (e * statin_{ij}) + u_{2i} + e_{2ij}$$

$$HDL\ cholesterol_{ij} = a_3 + b_3 age_{ij} + c_3 age_{ij}^2 + u_{3i} + e_{3ij}$$

$$BMI_{ij} = a_4 + b_4 age_{ij} + c_4 age_{ij}^2 + u_{4i} + e_{4ij}$$

$$\text{Where} \begin{bmatrix} u_{1i} \\ u_{2i} \\ u_{3i} \\ u_{4i} \end{bmatrix} \sim multivariate\ normal \left(\begin{bmatrix} 0 \\ 0 \\ 0 \\ 0 \end{bmatrix}, \begin{bmatrix} \sigma_1^2 & \sigma_{12} & \sigma_{13} & \sigma_{14} \\ \sigma_{12} & \sigma_2^2 & \sigma_{23} & \sigma_{24} \\ \sigma_{13} & \sigma_{23} & \sigma_3^2 & \sigma_{34} \\ \sigma_{14} & \sigma_{24} & \sigma_{34} & \sigma_4^2 \end{bmatrix} \right)$$

$$\text{And} \begin{bmatrix} e_{1ij} \\ e_{2ij} \\ e_{3ij} \\ e_{4ij} \end{bmatrix} \sim multivariate\ normal \left(\begin{bmatrix} 0 \\ 0 \\ 0 \\ 0 \end{bmatrix}, \begin{bmatrix} \sigma_{e1}^2 & 0 & 0 & 0 \\ 0 & \sigma_{e2}^2 & 0 & 0 \\ 0 & 0 & \sigma_{e3}^2 & 0 \\ 0 & 0 & 0 & \sigma_{e4}^2 \end{bmatrix} \right)$$

Where u_{1i} to u_{4i} represent the random intercepts but are correlated between risk factors. e_{1ij} to e_{4ij} represents the uncorrelated residual errors for each risk factor.

A mixed effects model was chosen to take into account the sporadic nature of electronic health records, as well as being able to model the intra-correlations between each risk factor. In addition, the model only needs a minimum of one recorded measurement of any one risk factor to estimate all four of the risk factors.

The model assumes that all risk factors jointly follow a multivariate normal distribution. Inference based from the multivariate normal distribution may often be reasonable even if the multivariate normality does not hold, especially in the context of imputation of missing data[53] and regression calibration[54,55].

Data S2.

Rescaling of prioritisation tool and formal risk assessment tool risks for population health modelling.

Our aim was to validate each prioritisation tool to estimate the health impact in a general population in England. We used UK Biobank due to its availability of detailed measurements at baseline, which was used to estimate a 10-year formal assessment risk, but also genetic data and linked historical primary care records necessary for the formal assessment model using conventional risk factors and PRS, and the prioritisation tools derived using primary care records and/or PRS. The breadth of the data allowed for a direct comparison of each prioritisation tool in the same individuals.

However, UKB participants have been shown to be healthier than the general population both in terms of risk factor levels and CVD incidence rates. Deriving and modelling the health impact of all prioritisation tools and formal risk tools in UK Biobank without adjustments would lead to a biased distribution of 10-year risks estimated, with the distribution of risks being skewed to the right and be narrow relative to the distribution observed in the general population. To more accurately use UK Biobank for population health modelling, the distribution of 10-year risks estimated were rescaled.

Rescaling was completed for each tool and by sex, using methods similar to those previously described[30], and allowed the mean level of predicted risks based on UKB data to match what was observed in CPRD. We used sex-specific mean risk factor levels calculated from the Clinical Practice Research Datalink (CPRD) between the years 2014 and 2019 within 5-year age groups to estimate the predicted risk in the general population by fitting the average level risk factors into the published QRISK2 risk model **(Table S2).** This allows us to calculate scaling factors to rescale each prioritisation tool and formal risk assessment model to have a distribution similar to what would be expected in the general population.

A linear model was fit within each tool and by sex to relate the observed risk (θ_{obs}) and predicted risk (θ_{pred}) estimated for each 5-year age group (c_s):

$$\log_e(-\log_e(1-\theta_{obs,c_s})) = \beta_0 + \beta_1 \times \log_e(-\log_e(1-\theta_{pred,c_s}))$$

The estimated β_0 and β_1 were then used as scaling factors to rescale each individual's original 10-year risk ($\theta_{pred,i}$) to give a new rescaled estimate $\theta_{newpred,i}$:

$$\theta_{newpred,i} = 1 - \exp(-\exp(\beta_0 + \beta_1 \times \log_e(-\log_e(1 - \theta_{pred,i}))))$$

Table S1. Code list used to define cardiovascular disease.

Endpoint	ICD-10 code
Angina pectoris	I20
Myocardial infarction	I21, I22, I23
Coronary disease non- myocardial infarction	I24, I25
Ischemic stroke	I63
Unclassified stroke	I64

HES data available covered hospital admissions. Death registries provided data on deaths, with both primary and contributory causes of death coded in ICD-10

Table S2. Age- and sex-specific mean risk factor levels using records from 870,486 individuals in the CPRD database.

	Men						Women					
Age group	40-44	45-49	50-54	55-59	60-64	65-69	40-44	45-49	50-54	55-59	60-64	65-69
Ethnicity — White, (%)	89.4	91.8	93.6	94.7	96	97.3	88.7	90.8	92.9	94.1	95.6	96.9
Townsend, mean*	-1.5	-1.5	-1.5	-1.5	-1.5	-1.5	-1.5	-1.5	-1.5	-1.5	-1.5	-1.5
Systolic blood pressure — mmHg, mean	129.5	131.2	132.8	134.4	135.8	136.5	121.0	124.3	127.5	130.0	132.6	134.8
Total cholesterol — mmol/litre, mean	5.18	5.26	5.31	5.27	5.22	5.13	4.85	5.07	5.36	5.61	5.70	5.70
HDL cholesterol — mmol/litre, mean	1.27	1.29	1.32	1.34	1.38	1.4	1.53	1.57	1.64	1.68	1.69	1.71
BMI – kg/m2, mean	28.2	28.6	28.5	28.3	27.9	27.4	27.8	28.2	28.2	28.1	27.7	27.2
Current/ever smoker, (%)	49.2	47.9	45.4	40.4	33.8	27.1	42.0	43.1	43.3	39.9	32.2	26.7
History of diabetes, (%)	6.0	7.0	8.3	10.2	11.6	13.4	10.1	10.5	11.0	11.3	11.7	12.6
Blood pressure-lowering medication prescriptions, (%)	6.9	9.9	13.7	18.7	24.2	30.5	12.3	16	20.5	25.6	30.0	35.9
Family history, (%)	6.1	7.0	7.5	7.6	7.0	6.7	7.4	0.9	9.7	10.4	10.4	9.8
Chronic kidney disease (4/5), (%)	0.2	0.1	0.2	0.2	0.2	0.3	0.2	0.2	0.2	0.2	0.3	0.3
Rheumatoid arthritis, (%)	0.4	0.6	0.7	0.8	1.1	1.2	1.1	1.4	1.6	2.0	2.5	2.7
Atrial fibrillation (%)	0.3	0.5	0.8	1.2	1.9	3.0	0.2	0.2	0.4	0.6	0.9	1.5
Coronary artery disease PRS†	-1.07	-1.10	-1.12	-1.14	-1.17	-1.17	-1.08	-1.10	-1.11	-1.13	-1.14	-1.15
Stroke PRS†	1.59	1.58	1.57	1.56	1.154	1.54	1.59	1.58	1.57	1.56	1.55	1.55

Abbreviations: BMI, body mass index; HDL cholesterol, high-density lipoprotein cholesterol; PRS, polygenic risk score.
*A mean Townsend score of -1.5 from UK Biobank due to insufficient data.
† Mean PRS values from UK Biobank were used due to lack of genetic data in CPRD.

Table S3. Age- and sex-specific crude 10-year cardiovascular disease incidence rates using records from 870,486 individuals in the CPRD database.

	Men						Women					
Age group	40-44	45-49	50-54	55-59	60-64	65-69	40-44	45-49	50-54	55-59	60-64	65-69
Crude incidence rate per 1,000 with at least at least one primary care record of systolic blood pressure, cholesterol, or BMI	18.2	34.4	58.1	86.6	112.3	144.6	11.5	18.0	28.6	39.0	58.1	73.3
Crude incidence rate per 1,000 including those without at least one primary care record of systolic blood pressure, cholesterol, or BMI	17.4	33.0	56.9	83.9	109.7	142.6	11.4	17.8	28.3	39.1	58.0	72.8

Abbreviations: BMI, body mass index.

Table S4. Age- and sex-specific prioritisation thresholds chosen for population health modelling.

	**Prioritisation using primary care records**	**Prioritisation using PRS**		**Prioritisation using PRS and primary care records**	
Age group	5% FNR prioritisation threshold, %	5% FNR prioritisation threshold, %	Equivalent events prioritisation threshold, %	5% FNR prioritisation threshold, %	Equivalent events prioritisation threshold, %
**Men**					
40-49	3.4%	3.3%	3.5%	3.9%	4.5%
50-59	6.5%	6.3%	6.5%	6.6%	7.4%
60-69	10.0%	10.6%	10.8%	9.6%	10.4%
**Women**					
40-49	3.0%	1.8%	1.9%	2.9%	4.2%
50-59	4.3%	3.6%	3.8%	4.5%	6.0%
60-69	6.9%	7.6%	7.9%	7.0%	8.6%

Abbreviations: FNR, false negative rate; PRS, polygenic risk score

Age group and sex specific 5% FNR prioritisation thresholds were defined as the level such that the expected false negative rate of the formal risk assessment is controlled to be 5%. The prioritisation thresholds were chosen by first, ranking the estimated 10-year CVD risks from each prioritisation tool amongst individuals with a future CVD event. The FNR threshold was selected as the maximum estimated risk such that 5% of individuals with a future event would not be prioritised (i.e. were lower than the threshold).

Age group and sex specific 'equivalent events prioritisation thresholds', when prioritising using PRS or PRS and primary care records tool, were chosen such that the number of events identified would be similar to if prioritising with primary care records using a 5% FNR prioritisation threshold.

Table S5. **The RECORD statement[37]– checklist of items, extended from the STROBE statement, that should be reported in observational studies using routinely collected health data.**

	Item No.	STROBE items	Location in manuscript where items are reported	RECORD items	Location in manuscript where items are reported
**Title and abstract**					
	1	(a) Indicate the study's design with a commonly used term in the title or the abstract (b) Provide in the abstract an informative and balanced summary of what was done and what was found	3	RECORD 1.1: The type of data used should be specified in the title or abstract. When possible, the name of the databases used should be included.<br><br>RECORD 1.2: If applicable, the geographic region and timeframe within which the study took place should be reported in the title or abstract.<br><br>RECORD 1.3: If linkage between databases was conducted for the study, this should be clearly stated in the title or abstract.	3<br><br>3

					3
**Introduction**					
Background rationale	2	Explain the scientific background and rationale for the investigation being reported	5		
Objectives	3	State specific objectives, including any prespecified hypotheses	5		
**Methods**					
Study Design	4	Present key elements of study design early in the paper	6		
Setting	5	Describe the setting, locations, and relevant dates, including periods of recruitment, exposure, follow-up, and data collection	6		
Participants	6	*(a) Cohort study* - Give the eligibility criteria, and the sources and methods of selection of participants. Describe methods of follow-up<br>*Case-control study* - Give the eligibility criteria, and the sources and methods of case ascertainment and control selection. Give the rationale for the choice of cases and controls<br>*Cross-sectional study* - Give the eligibility criteria, and the sources and methods of selection of participants	6	RECORD 6.1: The methods of study population selection (such as codes or algorithms used to identify subjects) should be listed in detail. If this is not possible, an explanation should be provided.<br><br>RECORD 6.2: Any validation studies of the codes or algorithms used to select the population should be referenced. If validation was conducted for this study and not published elsewhere, detailed methods and results should be provided.	7

		(b) Cohort study - For matched studies, give matching criteria and number of exposed and unexposed<br>*Case-control study* - For matched studies, give matching criteria and the number of controls per case		RECORD 6.3: If the study involved linkage of databases, consider use of a flow diagram or other graphical display to demonstrate the data linkage process, including the number of individuals with linked data at each stage.	7<br><br>Figure S2
Variables	7	Clearly define all outcomes, exposures, predictors, potential confounders, and effect modifiers. Give diagnostic criteria, if applicable.	7	RECORD 7.1: A complete list of codes and algorithms used to classify exposures, outcomes, confounders, and effect modifiers should be provided. If these cannot be reported, an explanation should be provided.	7, Table S 1
Data sources/ measurement	8	For each variable of interest, give sources of data and details of	7		

		methods of assessment (measurement).<br>Describe comparability of assessment methods if there is more than one group			
Bias	9	Describe any efforts to address potential sources of bias	8		
Study size	10	Explain how the study size was arrived at	Supplementary figures 1-2		
Quantitative variables	11	Explain how quantitative variables were handled in the analyses. If applicable, describe which groupings were chosen, and why	7-8		
Statistical methods	12	(a) Describe all statistical methods, including those used to control for confounding<br>(b) Describe any methods used to examine subgroups and interactions<br>(c) Explain how missing data were addressed<br>(d) *Cohort study* - If applicable, explain how loss to follow-up was addressed<br>*Case-control study* - If applicable, explain how matching of cases and controls was addressed<br>*Cross-sectional study* - If applicable, describe analytical methods taking account of sampling strategy<br>(e) Describe any sensitivity analyses	7-9		

Data access and cleaning methods		N/A		RECORD 12.1: Authors should describe the extent to which the investigators had access to the database population used to create the study population.<br><br>RECORD 12.2: Authors should provide information on the data cleaning methods used in the study.	6<br><br>6, Supplementary figures 1-2
Linkage		N/A		RECORD 12.3: State whether the study included person-level, institutional-level, or other data linkage across two or more databases. The methods of linkage and methods of linkage quality evaluation should be provided.	7
**Results**					
Participants	13	(a) Report the numbers of individuals at each stage of the study (*e.g.*, numbers potentially eligible, examined for eligibility, confirmed eligible, included in the study, completing follow-up, and analysed)	10, Supplementary figures 1-2	RECORD 13.1: Describe in detail the selection of the persons included in the study (*i.e.,* study population selection) including filtering based on data quality, data availability and linkage. The selection of included persons can be described	6, Supplementary figures 1-2

		(b) Give reasons for non-participation at each stage.<br>(c) Consider use of a flow diagram		in the text and/or by means of the study flow diagram.	
Descriptive data	14	(a) Give characteristics of study participants (*e.g.*, demographic, clinical, social) and information on exposures and potential confounders<br>(b) Indicate the number of participants with missing data for each variable of interest<br>(c) *Cohort study* - summarise follow-up time (*e.g.*, average and total amount)	Table 1		
Outcome data	15	*Cohort study* - Report numbers of outcome events or summary measures over time<br>*Case-control study* - Report numbers in each exposure category, or summary measures of exposure<br>*Cross-sectional study* - Report numbers of outcome events or summary measures	10		
Main results	16	(a) Give unadjusted estimates and, if applicable, confounder-adjusted estimates and their precision (e.g., 95% confidence interval). Make clear which confounders were adjusted for and why they were included			

		(b) Report category boundaries when continuous variables were categorized<br>(c) If relevant, consider translating estimates of relative risk into absolute risk for a meaningful time period	Table S 4		
Other analyses	17	Report other analyses done—e.g., analyses of subgroups and interactions, and sensitivity analyses	11-12, Table 4, Supplementary figures 5-6		
**Discussion**					
Key results	18	Summarise key results with reference to study objectives	13		
Limitations	19	Discuss limitations of the study, taking into account sources of potential bias or imprecision. Discuss both direction and magnitude of any potential bias	14	RECORD 19.1: Discuss the implications of using data that were not created or collected to answer the specific research question(s). Include discussion of misclassification bias, unmeasured confounding, missing data, and changing eligibility over time, as they pertain to the study being reported.	14
Interpretation	20	Give a cautious overall interpretation of results considering objectives, limitations, multiplicity of analyses, results from similar studies, and other relevant evidence	13		
Generalisability	21	Discuss the generalisability (external validity) of the study results	14-15		

**Other Information**					
Funding	22	Give the source of funding and the role of the funders for the present study and, if applicable, for the original study on which the present article is based	16		
Accessibility of protocol, raw data, and programming code		N/A		RECORD 22.1: Authors should provide information on how to access any supplemental information such as the study protocol, raw data, or programming code.	17

Table S6. Hazard ratios (95% confidence intervals) for the prioritisation and formal risk assessment tools derived using 44.184 men in UK Biobank.

**Risk factor**	Primary care records prioritisation tool	PRS + age prioritisation tool	PRS + primary care records prioritisation tool	Conventional risk factor formal assessment tool	Conventional risk factor + PRS formal assessment tool
Age – per year increase	1.071 (1.060, 1.083)	1.070 (1.062, 1.078)	1.075 (1.063, 1.086)	1.072 (1.060, 1.083)	1.075 (1.064, 1.087)
Ethnicity – non-White	0.946 (0.680, 1.316)	NA	0.464 (0.324, 0.665)	0.910 (0.654, 1.266)	0.463 (0.324, 0.663)
Townsend	1.032 (1.012, 1.052)	NA	1.030 (1.010, 1.050)	1.029 (1.010, 1.049)	1.028 (1.008, 1.048)
Smoking status – current/ever smoker	2.060 (1.765, 2.404)	NA	2.028 (1.737, 2.367)	1.979 (1.704, 2.298)	1.957 (1.685, 2.272)
Diabetes status - Yes	1.066 (0.548, 2.074)	NA	1.040 (0.535, 2.020)	1.524 (0.968, 2.400)	1.486 (0.943, 2.341)
Chronic kidney disease (stages 4/5)	2.777 (1.149, 6.709)	NA	2.691 (1.113, 6.504)	2.699 (1.118, 6.516)	2.655 (1.100, 6.412)
History of atrial fibrillation - Yes	0.662 (0.320, 1.368)	NA	0.651 (0.316, 1.342)	1.740 (0.614, 4.929)	1.666 (0.599, 4.631)
Anti-hypertensive medication - Yes	1.087 (0.912, 1.294)	NA	1.068 (0.897, 1.272)	1.203 (1.006, 1.439)	1.169 (0.978, 1.398)
Rheumatoid arthritis – Yes	0.624 (0.201, 1.940)	NA	0.625 (0.201, 1.942)	1.614 (1.047, 2.488)	1.562 (1.013, 2.408)
Family history of CVD – Yes	1.252 (0.941, 1.666)	NA	1.169 (0.878, 1.556)	1.254 (0.942, 1.668)	1.186 (0.891, 1.579)
Total cholesterol – per mmol/litre increase	1.571 (1.386, 1.780)	NA	1.503 (1.326, 1.705)	1.303 (1.231, 1.380)	1.276 (1.205, 1.351)
HDL – per mmol/litre increase	0.373 (0.256, 0.543)	NA	0.398 (0.274, 0.579)	0.411 (0.330, 0.511)	0.424 (0.341, 0.527)
Systolic blood pressure – per mmHg increase	1.025 (1.017, 1.034)	NA	1.023 (1.014, 1.032)	1.013 (1.009, 1.016)	1.012 (1.008, 1.015)
BMI – per kg/m2 increase	1.008 (0.987, 1.030)	NA	1.007 (0.986, 1.029)	1.017 (1.002, 1.033)	1.015 (1.000, 1.031)
CAD PRS – per SD increase	NA	1.299 (1.221, 1.381)	1.312 (1.231, 1.397)	NA	1.299 (1.219, 1.384)
Stroke PRS – per SD increase	NA	1.123 (1.059, 1.192)	1.162 (1.090, 1.239)	NA	1.150 (1.078, 1.226)
Age * BMI	1.000 (0.997, 1.002)	NA	1.000 (0.997, 1.002)	1.000 (0.998, 1.002)	1.000 (0.998, 1.002)
Age * Townsend	0.998 (0.996, 1.001)	NA	0.998 (0.995, 1.000)	0.998 (0.996, 1.001)	0.998 (0.996, 1.001)
Age * Systolic blood pressure	0.999 (0.998, 1.000)	NA	0.999 (0.998, 1.000)	1.000 (0.999, 1.000)	1.000 (0.999, 1.000)
Age * Family history of CVD	0.991 (0.954, 1.030)	NA	0.993 (0.955, 1.032)	0.994 (0.957, 1.032)	0.996 (0.959, 1.035)
Age * Smoking status	0.984 (0.964, 1.005)	NA	0.985 (0.965, 1.006)	0.981 (0.962, 1.000)	0.982 (0.963, 1.002)
Age * Anti-hypertensive medication	0.994 (0.972, 1.017)	NA	0.993 (0.971, 1.016)	0.984 (0.962, 1.008)	0.983 (0.961, 1.007)
Age * Diabetes status	1.069 (0.988, 1.158)	NA	1.070 (0.988, 1.158)	1.016 (0.961, 1.075)	1.018 (0.962, 1.077)
Age * History of atrial fibrillation	1.060 (0.968, 1.161)	NA	1.058 (0.966, 1.158)	1.026 (0.905, 1.164)	1.024 (0.906, 1.157)
Baseline survival estimate at 10 years	0.9668333	0.9670151	0.9672866	0.9777007	0.977394

Abbreviations: BMI, body mass index; CAD, coronary artery disease; CVD, cardiovascular disease; HDL cholesterol, high-density lipoprotein cholesterol; PRS, polygenic risk score; SD, standard deviation

Table S7. Hazard ratios (95% confidence intervals) for the prioritisation and formal risk assessment tools derived using 64,501 women in UK Biobank.

**Rick factor**	Primary care records prioritisation tool	PRS + age prioritisation tool	PRS + primary care records prioritisation tool	Conventional risk factor formal assessment tool	Conventional risk factor + PRS formal assessment tool
Age – per year increase	1.065 (1.045, 1.085)	1.094 (1.081, 1.107)	1.067 (1.048, 1.087)	1.076 (1.059, 1.094)	1.078 (1.061, 1.096)
Ethnicity – non-White	0.936 (0.573, 1.530)	NA	0.578 (0.338, 0.988)	0.872 (0.533, 1.426)	0.562 (0.329, 0.962)
Townsend	1.068 (1.038, 1.100)	NA	1.067 (1.036, 1.098)	1.064 (1.034, 1.096)	1.063 (1.032, 1.094)
Smoking status – current/ever smoker	2.695 (2.134, 3.405)	NA	2.662 (2.107, 3.362)	2.528 (2.003, 3.190)	2.502 (1.983, 3.157)
Diabetes status – History or	1.770 (0.811, 3.861)	NA	1.771 (0.812, 3.865)	1.501 (0.670, 3.363)	1.479 (0.659, 3.319)
Chronic kidney disease (stages 4/5)	0.841 (0.118, 5.993)	NA	0.795 (0.111, 5.667)	0.735 (0.102, 5.290)	0.695 (0.096, 5.021)
History of atrial fibrillation - Yes	0.874 (0.445, 1.717)	NA	0.881 (0.45, 1.726)	0.062 (0.000, 19.868)	0.064 (0.000, 21.315)
Anti-hypertensive medication - Yes	0.995 (0.771, 1.286)	NA	0.982 (0.76, 1.269)	1.658 (1.296, 2.123)	1.622 (1.267, 2.076)
Rheumatoid arthritis – Yes	1.934 (0.917, 4.077)	NA	1.921 (0.911, 4.05)	1.479 (0.924, 2.367)	1.495 (0.934, 2.393)
Family history of CVD – Yes	1.124 (0.734, 1.722)	NA	1.095 (0.714, 1.677)	1.097 (0.716, 1.679)	1.074 (0.701, 1.645)
Total cholesterol – per mmol/litre increase	1.406 (1.181, 1.675)	NA	1.369 (1.149, 1.631)	1.227 (1.137, 1.324)	1.210 (1.121, 1.307)
HDL – per mmol/litre increase	0.365 (0.238, 0.559)	NA	0.375 (0.245, 0.575)	0.540 (0.420, 0.694)	0.547 (0.425, 0.703)
Systolic blood pressure – per mmHg increase	1.038 (1.026, 1.049)	NA	1.036 (1.025, 1.048)	1.015 (1.010, 1.020)	1.014 (1.009, 1.019)
BMI – per kg/m2 increase	0.990 (0.966, 1.014)	NA	0.990 (0.966, 1.014)	1.011 (0.994, 1.030)	1.011 (0.993, 1.029)
CAD PRS – per SD increase	NA	1.178 (1.079, 1.286)	1.165 (1.064, 1.274)	NA	1.152 (1.053, 1.260)
Stroke PRS – per SD increase	NA	1.117 (1.025, 1.217)	1.115 (1.016, 1.225)	NA	1.106 (1.008, 1.215)
Age * BMI	1.002 (0.999, 1.004)	NA	1.002 (0.999, 1.004)	1.001 (0.999, 1.003)	1.001 (0.999, 1.003)
Age * Townsend	0.998 (0.994, 1.002)	NA	0.998 (0.994, 1.002)	0.998 (0.994, 1.001)	0.998 (0.994, 1.001)
Age * Systolic blood pressure	0.999 (0.998, 1.000)	NA	0.999 (0.998, 1.000)	1.000 (0.999, 1.000)	1.000 (0.999, 1.001)
Age * Family history of CVD	0.974 (0.916, 1.036)	NA	0.974 (0.916, 1.036)	0.977 (0.920, 1.037)	0.977 (0.920, 1.037)
Age * Smoking status	1.009 (0.977, 1.042)	NA	1.009 (0.977, 1.042)	1.019 (0.987, 1.051)	1.018 (0.987, 1.051)
Age * Anti-hypertensive medication	1.005 (0.972, 1.040)	NA	1.006 (0.972, 1.040)	0.975 (0.944, 1.007)	0.975 (0.944, 1.008)
Age * Diabetes status	1.005 (0.904, 1.117)	NA	1.006 (0.905, 1.118)	1.030 (0.928, 1.143)	1.032 (0.930, 1.146)
Age * History of atrial fibrillation	1.066 (0.979, 1.162)	NA	1.065 (0.978, 1.160)	1.551 (0.906, 2.653)	1.540 (0.898, 2.643)
Baseline survival estimate at 10 years	0.9859755	0.9896236	0.9862068	0.9915961	0.9915758

Abbreviations: BMI, body mass index; CAD, coronary artery disease; CVD, cardiovascular disease; HDL cholesterol, high-density lipoprotein cholesterol; PRS, polygenic risk score; SD, standard deviation

Table S8. Number needed to invite and screen to prevent one event, and number of events identified when prioritising with primary care records in a hypothetical population of 100,000 individuals in England.

			No prioritisation tool used: formal assessment with conventional risk factors used for all individuals			Prioritisation using primary care records, followed by formal assessment with conventional risk factors			
Age group	Participants	Expected number of events in 10 years	NNI (95% CI)	NNS (95% CI)	Number of events identified as high risk (%)	Participants prioritised (%)	NNI (95% CI)	NNS (95% CI)	Number of events identified as high risk (%)
**Men**									
40-49	18253	485	3033 (1986.4, 3692.0)	1517 (993.2, 1846.0)	120 (24.7%)	10126 (55.5%)	1530 (997.9, 1865.6)	841 (548.9, 1026.1)	120 (24.8%)
50-59	17391	1240	515 (463.8, 560.2)	257 (231.9, 280.1)	676 (54.5%)	12134 (69.8%)	339 (301.2, 368.3)	187 (165.7, 202.6)	651 (52.5%)
60-69	14356	1847	176 (171.0, 180.6)	88 (85.5, 90.3)	1629 (88.2%)	12517 (87.2%)	146 (140.8, 149.8)	80 (77.5, 82.4)	1564 (84.7%)
Total	50000	3573	412 (398.4, 426.3)	206 (199.2, 213.1)	2426 (67.9%)	34777 (69.6%)	271 (260.7, 281.1)	149 (143.4, 154.6)	2335 (65.3%)
**Women**									
40-49	18107	269	13462 (0.0, 19743.6)	6731 (0.0, 9871.8)	27 (10.0%)	3233 (17.9%)	2185 (0.0, 3192.3)	1202 (0.0, 1755.7)	27 (10.0%)
50-59	17282	577	2544 (1729.3, 3115.2)	1272 (864.6, 1557.6)	136 (23.6%)	8329 (48.2%)	1143 (778.9, 1403.4)	629 (428.4, 771.9)	132 (23.0%)
60-69	14611	962	457 (418.9, 488.1)	229 (209.4, 244.1)	639 (66.4%)	10459 (71.6%)	304 (277.4, 324.1)	167 (152.6, 178.3)	626 (65.1%)
Total	50000	1808	1248 (1151.8, 1336.1)	624 (575.9, 668.0)	801 (44.3%)	22021 (44.0%)	510 (470.8, 547.0)	280 (258.9, 300.8)	785 (43.4%)

Abbreviations: CI, confidence interval; NNS, number needed to screen; NNI, number needed to invite.

Age structure of hypothetical population extrapolated from Office for National Statistics, England, United Kingdom 2015. Expected events at 10 years based on extrapolation of incidence rates from CPRD, 2014-2019. Age group and sex specific prioritisation thresholds were defined as the level such that the expected false negative rate was controlled to be 5%. NNI and NNS assumes 50% statin compliance. NNI assumes a 50% invitation uptake if assessing without using prioritisation tool, and a 55% invitation uptake if assessing with using prioritisation tool. Formal assessment threshold set at 10% and prioritisation threshold set at equivalents to 5% false negative rate. Bootstrap 95% confidence intervals were calculated using 500 iterations.

Table S9. Number needed to invite and screen to prevent one event, and number of events identified when prioritising with PRS + age in a hypothetical population of 100,000 individuals in England.

			No prioritisation tool used: formal assessment with conventional risk factors + PRS used for all individuals			Prioritisation using PRS + age, followed by formal assessment with conventional risk factors + PRS			
Age group	Participants	Expected number of events in 10 years	NNI (95% CI)	NNS (95% CI)	Number of events identified as high risk (%)	Participants prioritised (%)	NNI (95% CI)	NNS (95% CI)	Number of events identified as high risk (%)
**Men**									
40-49	18253	485	2149 (1557.2, 2547.5)	1074 (778.6, 1273.8)	170 (35.1%)	13525 (74.1%)	1478 (1088.5, 1752.7)	813 (598.7, 964.0)	166 (34.3%)
50-59	17391	1240	494 (448.6, 534.4)	247 (224.3, 267.2)	705 (56.9%)	13456 (77.4%)	364 (328.9, 394.1)	200 (180.9, 216.7)	673 (54.3%)
60-69	14356	1847	181 (175.5, 186.7)	91 (87.7, 93.3)	1582 (85.7%)	12424 (86.5%)	149 (143.3, 153.9)	82 (78.8, 84.6)	1517 (82.1%)
Total	50000	3573	407 (393.0, 422.0)	204 (196.5, 211.0)	2457 (68.8%)	39405 (78.8%)	304 (292.3, 315.5)	167 (160.8, 173.5)	2356 (65.9%)
**Women**									
40-49	18107	269	11538 (0.0, 16718.6)	5769 (0.0, 8359.3)	31 (11.5%)	11442 (63.2%)	7733 (0.0, 11331.6)	4253 (0.0, 6232.4)	27 (10.0%)
50-59	17282	577	2077 (1455.0, 2472.3)	1038 (727.5, 1236.2)	166 (28.8%)	13590 (78.6%)	1516 (1050.2, 1820.7)	834 (577.6, 1001.4)	163 (28.3%)
60-69	14611	962	452 (418.5, 481.4)	226 (209.3, 240.7)	646 (67.2%)	12357 (84.6%)	360 (333.3, 383.7)	198 (183.3, 211.0)	623 (64.8%)
Total	50000	1808	1185 (1090.5, 1261.7)	592 (545.2, 630.8)	844 (46.7%)	37389 (74.8%)	836 (768.7, 893.4)	460 (422.8, 491.4)	813 (45.0%)

Abbreviations: CI, confidence interval; NNS, number needed to screen; NNI, number needed to invite; PRS, polygenic risk score.

Age structure of hypothetical population extrapolated from Office for National Statistics, England, United Kingdom 2015. Expected events at 10 years based on extrapolation of incidence rates from CPRD, 2014-2019. Age group and sex specific prioritisation thresholds were defined as the level such that the expected false negative rate was controlled to be 5%. NNI and NNS assumes 50% statin compliance. NNI assumes a 50% invitation uptake if assessing without using prioritisation tool, and a 55% invitation uptake if assessing with using prioritisation tool. Formal assessment threshold set at 10% and prioritisation threshold set at equivalents to 5% false negative rate. Bootstrap 95% confidence intervals were calculated using 500 iterations.

Table S10. Number needed to invite and screen to prevent one event, and number of events identified when prioritising with PRS and primary care records in a hypothetical population of 100,000 individuals in England.

			No prioritisation tool used: formal assessment with conventional risk factors + PRS used for all individuals			Prioritisation using PRS and primary care records, followed by formal assessment with conventional risk factors + PRS			
Age group	Participants	Expected number of events in 10 years	NNI (95% CI)	NNS (95% CI)	Number of events identified as high risk (%)	Participants prioritised (%)	NNI (95% CI)	NNS (95% CI)	Number of events identified as high risk (%)
**Men**									
40-49	18253	485	2149 (1557.2, 2547.5)	1074 (778.6, 1273.8)	170 (35.1%)	7930 (43.4%)	885 (640.1, 1051.0)	487 (352.1, 578.0)	163 (33.6%)
50-59	17391	1240	494 (448.6, 534.4)	247 (224.3, 267.2)	705 (56.9%)	10622 (61.1%)	288 (259.9, 312.3)	159 (143.0, 171.8)	670 (54.0%)
60-69	14356	1847	181 (175.5, 186.7)	91 (87.7, 93.3)	1582 (85.7%)	11436 (79.7%)	135 (130.4, 139.7)	75 (71.7, 76.9)	1535 (83.1%)
Total	50000	3573	407 (393.0, 422.0)	204 (196.5, 211.0)	2457 (68.8%)	29988 (60.0%)	230 (221.3, 239.5)	127 (121.7, 131.7)	2367 (66.3%)
**Women**									
40-49	18107	269	11538 (0.0, 16718.6)	5769 (0.0, 8359.3)	31 (11.5%)	3513 (19.4%)	2035 (0.0, 2953.9)	1119 (0.0, 1624.7)	31 (11.7%)
50-59	17282	577	2077 (1455.0, 2472.3)	1038 (727.5, 1236.2)	166 (28.8%)	7616 (44.1%)	867 (613.9, 1038.0)	477 (337.6, 570.9)	160 (27.7%)
60-69	14611	962	452 (418.5, 481.4)	226 (209.3, 240.7)	646 (67.2%)	9872 (67.6%)	283 (261.8, 301.7)	156 (144.0, 165.9)	634 (65.9%)
Total	50000	1808	1185 (1090.5, 1261.7)	592 (545.2, 630.8)	844 (46.7%)	21001 (42.0%)	463 (426.0, 495.6)	255 (234.3, 272.6)	825 (45.6%)

Abbreviations: CI, confidence interval; NNS, number needed to screen; NNI, number needed to invite; PRS, polygenic risk score.

Age structure of hypothetical population extrapolated from Office for National Statistics, England, United Kingdom 2015. Expected events at 10 years based on extrapolation of incidence rates from CPRD, 2014-2019. Age group and sex specific prioritisation thresholds were defined as the level such that the expected false negative rate was controlled to be 5%. NNI and NNS assumes 50% statin compliance. NNI assumes a 50% invitation uptake if assessing without using prioritisation tool, and a 55% invitation uptake if assessing with using prioritisation tool. Formal assessment threshold set at 10% and prioritisation threshold set at equivalents to 5% false negative rate. Bootstrap 95% confidence intervals were calculated using 500 iterations.

Table S11. Summary of number of individuals without primary care records in UK Biobank.

Sex	Age group	Individuals without at least one CVD risk factor in primary care record N (%)
Men	40-49	3851 (25.0%)
	50-59	2736 (14.8%)
	60-69	1734 (9.3%)
Women	40-49	2714 (14.0%)
	50-59	2415 (9.2%)
	60-69	1874 (7.2%)

Abbreviations: CVD, cardiovascular disease.

Prioritisation with primary care records requires at least one CVD risk factor of: systolic blood pressure, total cholesterol, HDL cholesterol and/or BMI.

Table S12. Number needed to invite and screen to prevent one event and number of events identified when prioritising using primary care records, including all individuals without a primary care record for any one of SBP, HDL, total cholesterol or BMI, in a hypothetical population of 100,000 individuals in England.

			No prioritisation tool used: formal assessment with conventional risk factors used for all individuals			Prioritisation using primary care records, followed by formal assessment with conventional risk factors			
Age group	Participants	Expected number of events in 10 years	NNI (95% CI)	NNS (95% CI)	Number of events identified as high risk (%)	Participants prioritised (%)	NNI (95% CI)	NNS (95% CI)	Number of events identified as high risk (%)
**Men**									
40-49	18253	465	3042 (2126.4, 3710.8)	1521 (1063.2, 1855.4)	120 (25.8%)	12154 (66.6%)	1842 (1301.8, 2245.1)	1012 (716.0, 1234.8)	120 (25.8%)
50-59	17391	1207	536 (487.7, 577.3)	268 (243.9, 288.6)	650 (53.9%)	12914 (74.3%)	374 (340.0, 404.5)	206 (187.0, 222.5)	628 (52.0%)
60-69	14356	1814	178 (173.6, 182.4)	89 (86.8, 91.2)	1611 (88.8%)	12688 (88.4%)	148 (144.2, 152.4)	82 (79.3, 83.8)	1551 (85.5%)
Total	50000	3486	420 (407.2, 433.8)	210 (203.6, 216.9)	2381 (68.3%)	37756 (75.5%)	298 (288.5, 308.9)	164 (158.7, 169.9)	2300 (66.0%)
**Women**									
40-49	18107	267	14268 (0.0, 20446.9)	7134 (0.0, 10223.4)	25 (9.4%)	5316 (29.4%)	3808 (0.0, 5459.1)	2094 (0.0, 3002.5)	25 (9.5%)
50-59	17282	575	2526 (1502.0, 3023.8)	1263 (751.0, 1511.9)	137 (23.8%)	9153 (53.0%)	1244 (731.9, 1501.7)	684 (402.5, 825.9)	134 (23.3%)
60-69	14611	957	464 (434.0, 494.8)	232 (217.0, 247.4)	629 (65.7%)	10760 (73.6%)	316 (295.8, 338.7)	174 (162.7, 186.3)	617 (64.5%)
Total	50000	1799	1264 (1167.6, 1350.6)	632 (583.8, 675.3)	792 (44.0%)	25229 (50.5%)	590 (543.1, 633.7)	324 (298.7, 348.5)	776 (43.2%)

Abbreviations: CI, confidence interval; HDL, high-density lipoprotein; NNI, number needed to invite; NNS, number needed to screen; SBP, systolic blood pressure.

Age structure of hypothetical population extrapolated from Office for National Statistics, England, United Kingdom 2015. Expected events at 10 years based on extrapolation of incidence rates from CPRD, 2014-2019. Age group and sex specific prioritisation thresholds were defined as the level such that the expected false negative rate was controlled to be 5%. NNI and NNS assumes 50% statin compliance. NNI assumes a 50% invitation uptake if assessing without using prioritisation tool, and a 55% invitation uptake if assessing with using prioritisation tool. Formal assessment threshold set at 10% and prioritisation threshold set at equivalents to 5% false negative rate. Bootstrap 95% confidence intervals were calculated using 500 iterations.

Table S13. Number need to invite and screen to prevent one event, and number of events identified when prioritising using PRS + age, including all individuals without a primary care record for any one of SBP, HDL, total cholesterol or BMI, in a hypothetical population of 100,000 individuals in England.

			**No prioritisation tool used: formal assessment with conventional risk factors + PRS used for all individuals**			**Prioritisation using PRS + age, followed by formal assessment with conventional risk factors + PRS**			
Age group	Participants	Expected number of events in 10 years	NNI (95% CI)	NNS (95% CI)	Number of events identified as high risk (%)	Participants prioritised (%)	NNI (95% CI)	NNS (95% CI)	Number of events identified as high risk (%)
**Men**									
40-49	18253	465	2282 (1717.6, 2656.7)	1141 (858.8, 1328.4)	160 (34.4%)	13287 (72.8%)	1534 (1150.5, 1802.9)	844 (632.8, 991.6)	157 (33.9%)
50-59	17391	1207	506 (460.6, 543.5)	253 (230.3, 271.7)	688 (57.0%)	13338 (76.7%)	368 (335.9, 397.3)	202 (184.8, 218.5)	658 (54.5%)
60-69	14356	1814	184 (178.1, 188.8)	92 (89.0, 94.4)	1563 (86.2%)	12393 (86.3%)	150 (145.4, 156.0)	82 (80.0, 85.8)	1495 (82.4%)
Total	50000	3486	414 (400.2, 427.8)	207 (200.1, 213.9)	2411 (69.2%)	39018 (78.0%)	308 (296.0, 316.7)	168 (162.8, 174.2)	2310 (66.3%)
**Women**									
40-49	18107	267	12230 (0.0, 17211.5)	6115 (0.0, 8605.7)	30 (11.2%)	11224 (62.0%)	8040 (0.0, 11532.0)	4422 (0.0, 6342.6)	25 (9.5%)
50-59	17282	575	2098 (1490.7, 2462.6)	1049 (745.3, 1231.3)	165 (28.7%)	13545 (78.4%)	1552 (1081.7, 1836.9)	854 (594.9, 1010.3)	159 (27.6%)
60-69	14611	957	460 (430.5, 489.6)	230 (215.3, 244.8)	634 (66.2%)	12341 (84.5%)	368 (341.3, 393.0)	202 (187.7, 216.1)	610 (63.8%)
Total	50000	1799	1206 (1110.0, 1293.8)	603 (555.0, 646.9)	829 (46.1%)	37110 (74.2%)	850 (783.2, 914.0)	468 (430.8, 502.7)	794 (44.1%)

Abbreviations: CI, confidence interval; HDL, high-density lipoprotein; NNI, number needed to invite; NNS, number needed to screen; PRS, polygenic risk score; SBP, systolic blood pressure.

Age structure of hypothetical population extrapolated from Office for National Statistics, England, United Kingdom 2015. Expected events at 10 years based on extrapolation of incidence rates from CPRD, 2014-2019. Age group and sex specific prioritisation thresholds were defined as the level such that the expected false negative rate was controlled to be 5%. NNI and NNS assumes 50% statin compliance. NNI assumes a 50% invitation uptake if assessing without using prioritisation tool, and a 55% invitation uptake if assessing with using prioritisation tool. Formal assessment threshold set at 10% and prioritisation threshold set at equivalents to 5% false negative rate. Bootstrap 95% confidence intervals were calculated using 500 iterations.

Table S14. Number need to invite and screen to prevent one event, and number of events identified when prioritising using PRS and primary care records, including all individuals without a primary care record for any one of SBP, HDL, total cholesterol or BMI, in a hypothetical population of 100,000 individuals in England.

			No prioritisation tool used: formal assessment with conventional risk factors + PRS used for all individuals			Prioritisation using PRS and primary care records, followed by formal assessment with conventional risk factors + PRS			
Age group	Participants	Expected number of events in 10 years	NNI (95% CI)	NNS (95% CI)	Number of events identified as high risk (%)	Participants prioritised (%)	NNI (95% CI)	NNS (95% CI)	Number of events identified as high risk (%)
**Men**									
40-49	18253	465	2282 (1717.6, 2656.7)	1141 (858.8, 1328.4)	160 (34.4%)	10505 (57.6%)	1232 (931.5, 1451.5)	678 (512.3, 798.3)	155 (33.3%)
50-59	17391	1207	506 (460.6, 543.5)	253 (230.3, 271.7)	688 (57.0%)	11626 (66.9%)	322 (290.4, 346.7)	176 (159.7, 190.7)	658 (54.5%)
60-69	14356	1814	184 (178.1, 188.8)	92 (89.0, 94.4)	1563 (86.2%)	11708 (81.6%)	140 (135.1, 144.4)	76 (74.3, 79.4)	1521 (83.8%)
Total	50000	3486	414 (400.2, 427.8)	207 (200.1, 213.9)	2411 (69.2%)	33840 (67.7%)	264 (254.5, 273.2)	144 (140.0, 150.3)	2334 (66.9%)
**Women**									
40-49	18107	267	12230 (0.0, 17211.5)	6115 (0.0, 8605.7)	30 (11.2%)	5557 (30.7%)	3412 (0.0, 4796.1)	1876 (0.0, 2637.9)	30 (11.1%)
50-59	17282	575	2098 (1490.7, 2462.6)	1049 (745.3, 1231.3)	165 (28.7%)	8506 (49.2%)	976 (674.4, 1143.8)	536 (370.9, 629.1)	159 (27.6%)
60-69	14611	957	460 (430.5, 489.6)	230 (215.3, 244.8)	634 (66.2%)	10215 (69.9%)	298 (278.5, 317.6)	164 (153.2, 174.7)	622 (65.0%)
Total	50000	1799	1206 (1110.0, 1293.8)	603 (555.0, 646.9)	829 (46.1%)	24278 (48.6%)	544 (499.8, 582.7)	300 (274.9, 320.5)	810 (45.0%)

Abbreviations: CI, confidence interval; HDL, high-density lipoprotein; NNI, number needed to invite; NNS, number needed to screen; PRS, polygenic risk score; SBP, systolic blood pressure.

Age structure of hypothetical population extrapolated from Office for National Statistics, England, United Kingdom 2015. Expected events at 10 years based on extrapolation of incidence rates from CPRD, 2014-2019. Age group and sex specific prioritisation thresholds were defined as the level such that the expected false negative rate was controlled to be 5%. NNI and NNS assumes 50% statin compliance. NNI assumes a 50% invitation uptake if assessing without using prioritisation tool, and a 55% invitation uptake if assessing with using prioritisation tool. Formal assessment threshold set at 10% and prioritisation threshold set at equivalents to 5% false negative rate. Bootstrap 95% confidence intervals were calculated using 500 iterations.

Table S15. Age- and sex-specific prioritisation thresholds chosen for population health modelling with 2.5% false negative rate prioritisation thresholds.

	**Prioritisation using primary care records**	**Prioritisation using PRS**	**Prioritisation using PRS and primary care records**
Age group	2.5% FNR prioritisation threshold, %	2.5% FNR prioritisation threshold, %	2.5% FNR prioritisation threshold, %
**Men**			
40-49	2.2%	2.7%	2.3%
50-59	4.3%	4.9%	4.1%
60-69	7.5%	8.3%	6.2%
**Women**			
40-49	1.7%	1.4%	1.6%
50-59	2.8%	3.0%	2.9%
60-69	4.5%	6.5%	4.3%

Abbreviations: FNR, false negative rate; PRS, polygenic risk score

Age group and sex specific 2.5% FNR prioritisation thresholds were defined as the level such that the expected false negative rate of the formal risk assessment is controlled to be 2.5%. The prioritisation thresholds were chosen by first, ranking the estimated 10-year CVD risks from each prioritisation tool amongst individuals with a future CVD event. The FNR threshold was selected as the maximum estimated risk such that 2.5% of individuals with a future event would not be prioritised (i.e. were lower than the threshold).

Table S16. Number needed to invite and screen to prevent one event, and number of events identified when prioritising with primary care records in a hypothetical population of 100,000 individuals in England, assuming a 5% formal risk assessment threshold.

			**No prioritisation tool used: formal assessment with conventional risk factors used for all individuals**			**Prioritisation using primary care records, followed by formal assessment with conventional risk factors**			
Age group	Participants	Expected number of events in 10 years	NNI (95% CI)	NNS (95% CI)	Number of events identified as high risk (%)	Participants prioritised (%)	NNI (95% CI)	NNS (95% CI)	Number of events identified as high risk (%)
**Men**									
40-49	18253	485	1063 (937.6, 1160.5)	532 (468.8, 580.3)	343 (70.7%)	14972 (82.0%)	810 (708.0, 888.0)	445 (389.4, 488.4)	336 (69.3%)
50-59	17391	1240	310 (300.4, 319.7)	155 (150.2, 159.8)	1122 (90.5%)	15891 (91.4%)	260 (251.2, 268.0)	143 (138.2, 147.4)	1113 (89.7%)
60-69	14356	1847	157 (155.5, 157.7)	78 (77.8, 78.8)	1831 (99.1%)	13873 (96.6%)	140 (137.7, 140.9)	77 (75.7, 77.5)	1808 (97.9%)
Total	50000	3573	303 (299.2, 308.0)	152 (149.6, 154.0)	3297 (92.3%)	44735 (89.5%)	250 (245.8, 254.1)	137 (135.2, 139.7)	3257 (91.2%)
**Women**									
40-49	18107	269	4251 (2235.2, 5417.1)	2126 (1117.6, 2708.6)	85 (31.6%)	8316 (45.9%)	1775 (936.4, 2263.8)	976 (515.0, 1245.1)	85 (31.7%)
50-59	17282	577	901 (798.7, 984.6)	450 (399.3, 492.3)	384 (66.6%)	13140 (76.0%)	634 (559.1, 694.9)	349 (307.5, 382.2)	377 (65.3%)
60-69	14611	962	321 (313.2, 327.7)	160 (156.6, 163.9)	911 (94.7%)	13626 (93.3%)	276 (267.9, 282.5)	152 (147.3, 155.4)	898 (93.4%)
Total	50000	1808	725 (697.2, 752.1)	362 (348.6, 376.0)	1380 (76.3%)	35082 (70.2%)	469 (449.4, 487.1)	258 (247.2, 267.9)	1361 (75.2%)

Abbreviations: CI, confidence interval; NNS, number needed to screen; NNI, number needed to invite.

Age structure of hypothetical population extrapolated from Office for National Statistics, England, United Kingdom 2015. Expected events at 10 years based on extrapolation of incidence rates from CPRD, 2014-2019. Age group and sex specific prioritisation thresholds were defined as the level such that the expected false negative rate was controlled to be 5%. NNI and NNS assumes 50% statin compliance. NNI assumes a 50% invitation uptake if assessing without using prioritisation tool, and a 55% invitation uptake if assessing with using prioritisation tool. Formal assessment threshold set at 5% and prioritisation thresholds set at equivalent to 2.5% false negative rate. Bootstrap 95% confidence intervals were calculated using 500 iterations.

Table S17. Number needed to invite and screen to prevent one event, and number of events identified when prioritising with PRS + age in a hypothetical population of 100,000 individuals in England, assuming a 5% formal risk assessment threshold.

			No prioritisation tool used: formal assessment with conventional risk factors + PRS used for all individuals			Prioritisation using PRS + age, followed by formal assessment with conventional risk factors + PRS			
Age group	Participants	Expected number of events in 10 years	NNI (95% CI)	NNS (95% CI)	Number of events identified as high risk (%)	Participants prioritised (%)	NNI (95% CI)	NNS (95% CI)	Number of events identified as high risk (%)
**Men**									
40-49	18253	485	1063 (938.9, 1169.9)	532 (469.4, 585.0)	343 (70.7%)	15963 (87.5%)	863 (747.8, 955.3)	475 (411.3, 525.4)	336 (69.3%)
50-59	17391	1240	317 (306.4, 327.8)	159 (153.2, 163.9)	1097 (88.5%)	15932 (91.6%)	266 (256.9, 275.9)	147 (141.3, 151.7)	1087 (87.7%)
60-69	14356	1847	157 (155.7, 158.1)	79 (77.9, 79.1)	1829 (99.0%)	13800 (96.1%)	139 (137.6, 140.8)	77 (75.7, 77.5)	1800 (97.5%)
Total	50000	3573	306 (301.0, 311.5)	153 (150.5, 155.7)	3269 (91.5%)	45695 (91.4%)	258 (253.1, 262.5)	142 (139.2, 144.4)	3224 (90.2%)
**Women**									
40-49	18107	269	4487 (2184.7, 5762.6)	2244 (1092.3, 2881.3)	81 (30.1%)	15735 (86.9%)	3545 (1720.9, 4546.4)	1950 (946.5, 2500.5)	81 (30.0%)
50-59	17282	577	893 (788.5, 971.7)	446 (394.3, 485.9)	387 (67.1%)	15653 (90.6%)	755 (668.7, 826.2)	415 (367.8, 454.4)	377 (65.3%)
60-69	14611	962	326 (317.3, 334.4)	163 (158.7, 167.2)	896 (93.1%)	13869 (94.9%)	286 (276.5, 294.2)	158 (152.1, 161.8)	880 (91.5%)
Total	50000	1808	733 (704.6, 759.3)	367 (352.3, 379.6)	1364 (75.4%)	45257 (90.5%)	615 (591.0, 637.9)	338 (325.0, 350.9)	1338 (74.0%)

Abbreviations: CI, confidence interval; NNS, number needed to screen; NNI, number needed to invite; PRS, polygenic risk score.

Age structure of hypothetical population extrapolated from Office for National Statistics, England, United Kingdom 2015. Expected events at 10 years based on extrapolation of incidence rates from CPRD, 2014-2019. Age group and sex specific prioritisation thresholds were defined as the level such that the expected false negative rate was controlled to be 5%. NNI and NNS assumes 50% statin compliance. NNI assumes a 50% invitation uptake if assessing without using prioritisation tool, and a 55% invitation uptake if assessing with using prioritisation tool. Formal assessment threshold set at 5% and prioritisation thresholds set at equivalent to 2.5% false negative rate. Bootstrap 95% confidence intervals were calculated using 500 iterations.

Table S18. Number needed to invite and screen to prevent one event, and number of events identified when prioritising with PRS and primary care records in a hypothetical population of 100,000 individuals in England, assuming a 5% formal risk assessment threshold.

			No prioritisation tool used: formal assessment with conventional risk factors + PRS used for all individuals			Prioritisation using PRS and primary care records, followed by formal assessment with conventional risk factors + PRS			
Age group	Participants	Expected number of events in 10 years	NNI (95% CI)	NNS (95% CI)	Number of events identified as high risk (%)	Participants prioritised (%)	NNI (95% CI)	NNS (95% CI)	Number of events identified as high risk (%)
**Men**									
40-49	18253	485	1063 (938.9, 1169.9)	532 (469.4, 585.0)	343 (70.7%)	13310 (72.9%)	705 (624.0, 777.2)	388 (343.2, 427.5)	343 (70.8%)
50-59	17391	1240	317 (306.4, 327.8)	159 (153.2, 163.9)	1097 (88.5%)	14961 (86.0%)	251 (241.4, 259.8)	138 (132.8, 142.9)	1084 (87.4%)
60-69	14356	1847	157 (155.7, 158.1)	79 (77.9, 79.1)	1829 (99.0%)	13629 (94.9%)	136 (135.1, 137.7)	75 (74.3, 75.8)	1816 (98.3%)
Total	50000	3573	306 (301.0, 311.5)	153 (150.5, 155.7)	3269 (91.5%)	41899 (83.8%)	235 (230.7, 239.3)	129 (126.9, 131.6)	3243 (90.8%)
**Women**									
40-49	18107	269	4487 (2184.7, 5762.6)	2244 (1092.3, 2881.3)	81 (30.1%)	8708 (48.1%)	1962 (960.2, 2525.6)	1079 (528.1, 1389.1)	81 (30.0%)
50-59	17282	577	893 (788.5, 971.7)	446 (394.3, 485.9)	387 (67.1%)	12540 (72.6%)	610 (538.4, 665.9)	336 (296.1, 366.3)	374 (64.8%)
60-69	14611	962	326 (317.3, 334.4)	163 (158.7, 167.2)	896 (93.1%)	13376 (91.5%)	276 (267.5, 283.9)	152 (147.1, 156.2)	880 (91.5%)
Total	50000	1808	733 (704.6, 759.3)	367 (352.3, 379.6)	1364 (75.4%)	34623 (69.2%)	472 (451.1, 489.4)	259 (248.1, 269.2)	1335 (73.8%)

Abbreviations: CI, confidence interval; NNS, number needed to screen; NNI, number needed to invite; PRS, polygenic risk score.

Age structure of hypothetical population extrapolated from Office for National Statistics, England, United Kingdom 2015. Expected events at 10 years based on extrapolation of incidence rates from CPRD, 2014-2019. Age group and sex specific prioritisation thresholds were defined as the level such that the expected false negative rate was controlled to be 5%. NNI and NNS assumes 50% statin compliance. NNI assumes a 50% invitation uptake if assessing without using prioritisation tool, and a 55% invitation uptake if assessing with using prioritisation tool. Formal assessment threshold set at 5% and prioritisation threshold set at equivalents to 2.5% false negative rate. Bootstrap 95% confidence intervals were calculated using 500 iterations.

Figure S1. **Flowchart showing data sources used for model derivation for estimated risks and population health modelling.**

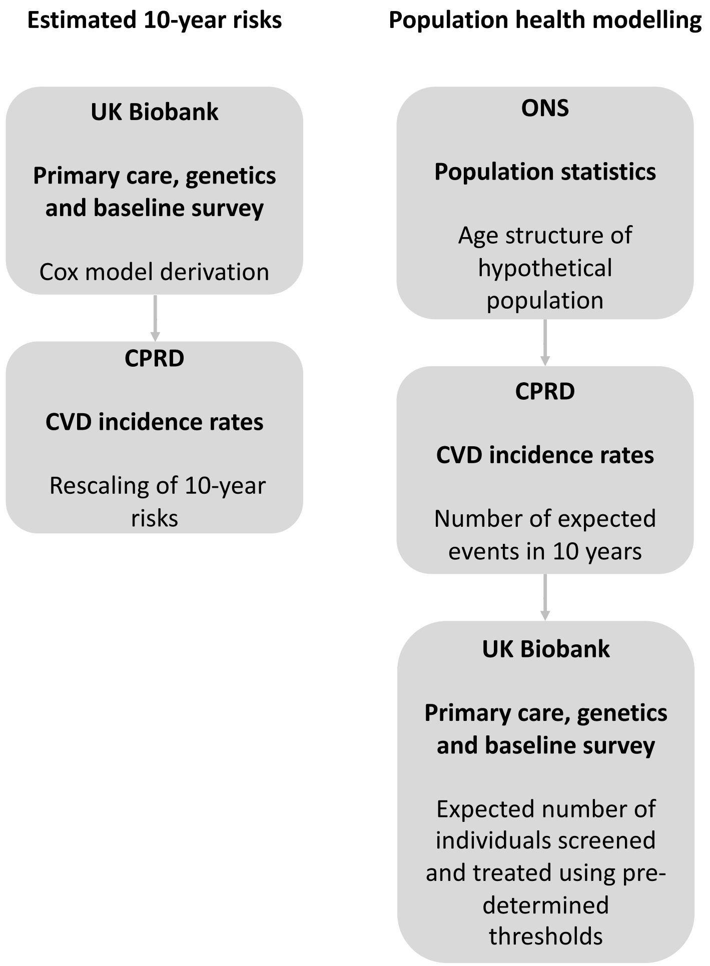

Abbreviations: CPRD, Clinical Practice Research Datalink; ONS, Office for National Statistics.

Figure S2. Flowchart showing selection of patient records for generating summary statistics from CPRD.

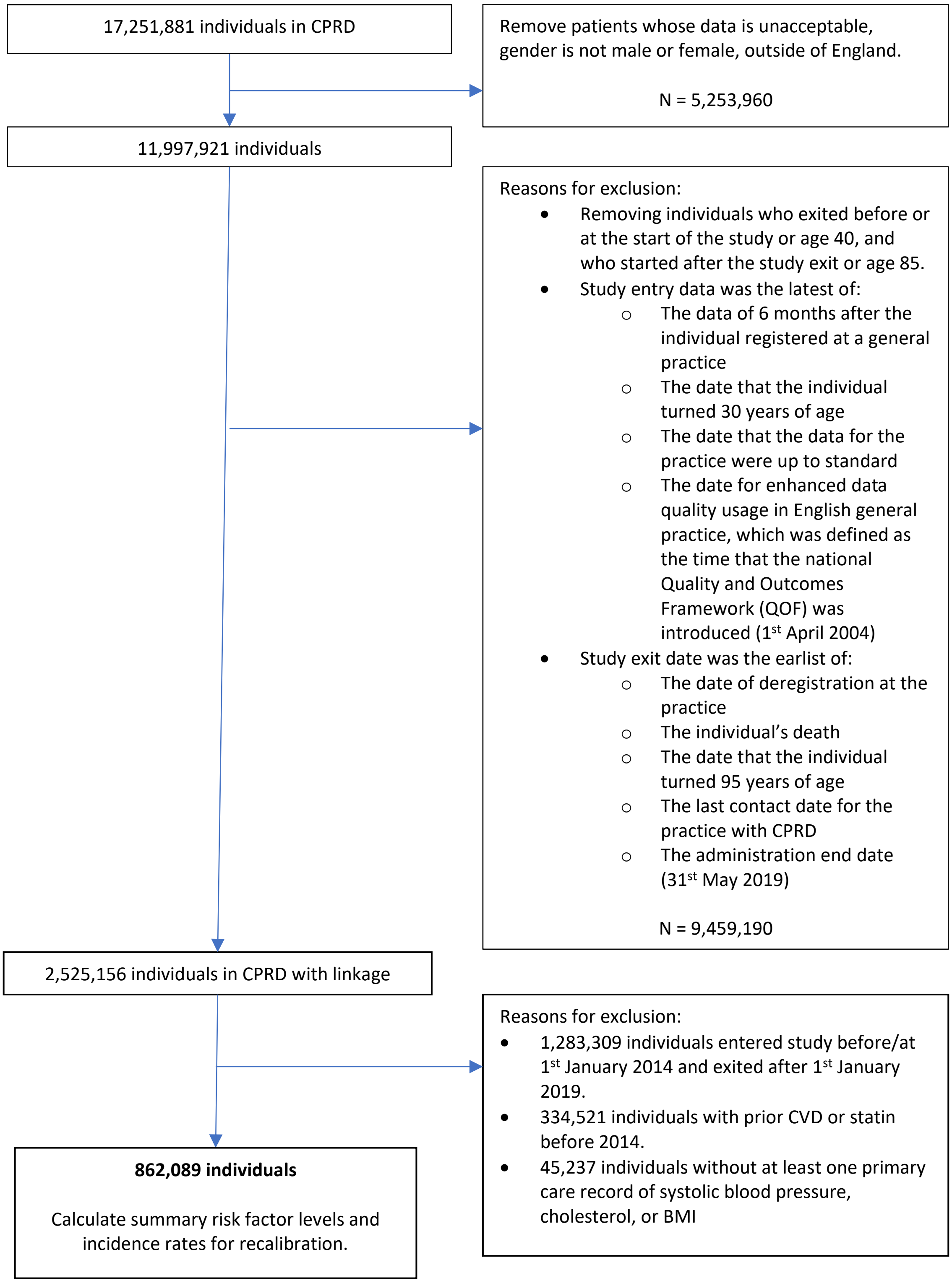

Abbreviations: BMI, body mass index; CVD, cardiovascular disease.

Highlighted in red were individuals without necessary primary care records to calculate incidence rates for sensitivity analyses and were included for sensitivity analysis incidence rates calculation.

Figure S3. **Illustration of UK Biobank data used in analysis.**

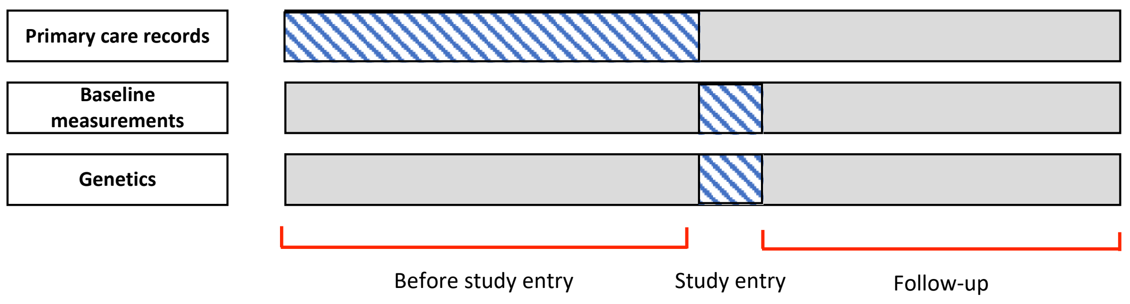

The UK Biobank baseline data was used to derive and calculate the formal CVD risk assessment. The genetics data was used to derive the polygenic risk scores and were taken at the same time (First UK Biobank survey). The retrospective primary care data were taken at different time points from 1st April 2004 until baseline survey.

Figure S4. Flowchart showing selection of patient records for derivation and population health modelling in UK Biobank.

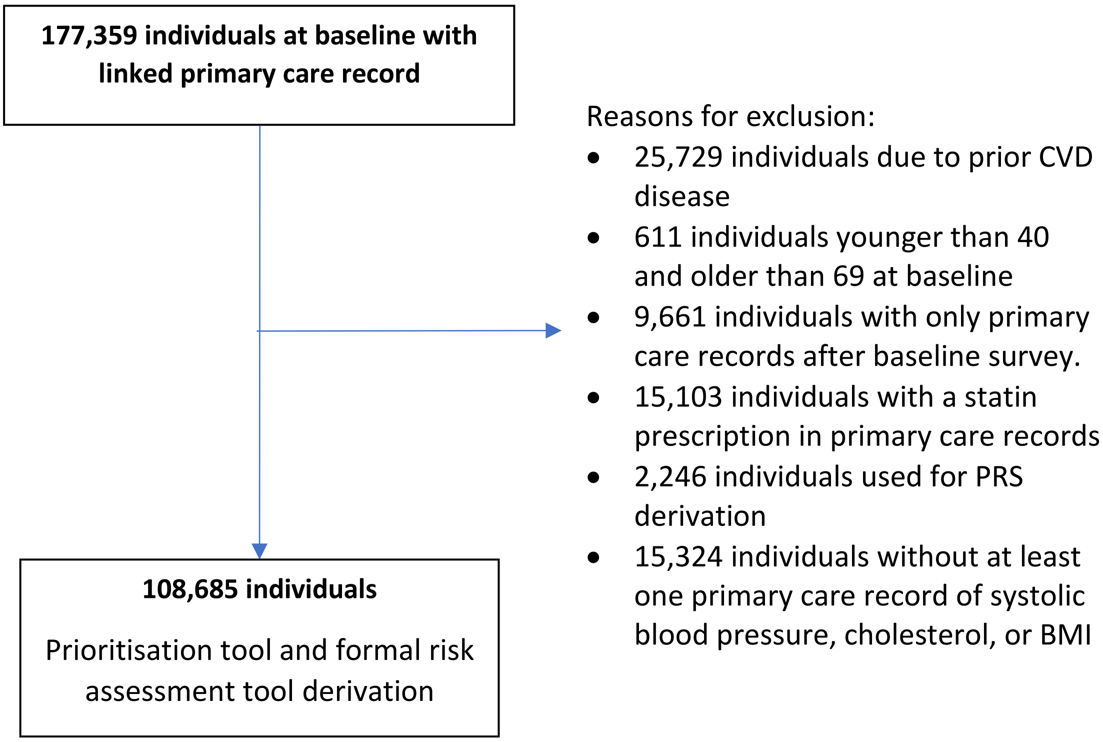

Abbreviations: BMI, body mass index; CVD, cardiovascular disease; PRS, polygenic risk score.

Highlighted in red are individuals without necessary primary care records for primary care based prioritisation tool that were formally assessed in sensitivity analysis.

Figure S5. Age group and sex specific distributions of rescaled 10-year risks for each prioritisation tool and formal assessment tool.

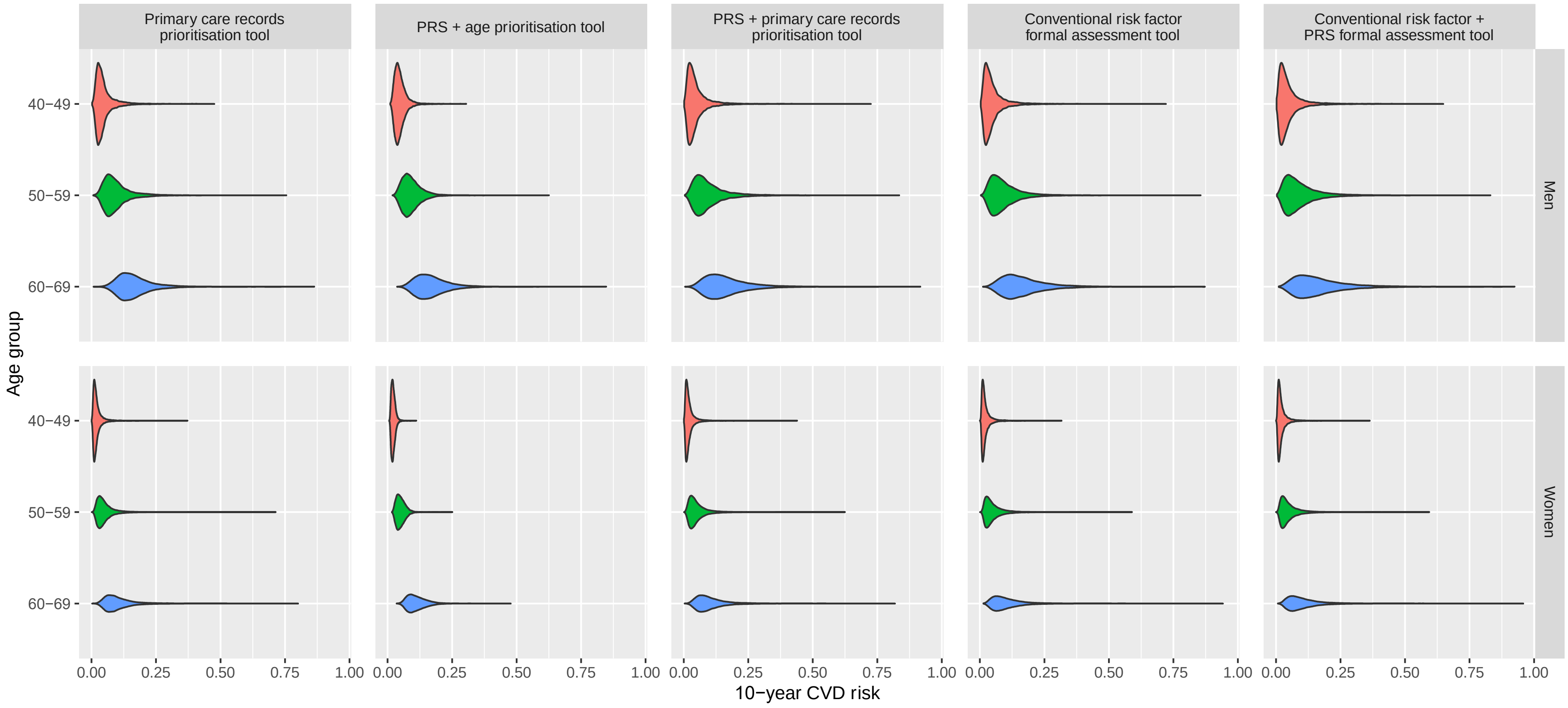

Figure S6. Number needed to invite and screen to prevent one event and number of events identified after prioritisation and formal assessment in a hypothetical population of 100,000 individuals in England, with prioritisation thresholds selected to identify the same number of events if prioritising with primary care records with prioritisation thresholds controlling the false negative rate to 5%.

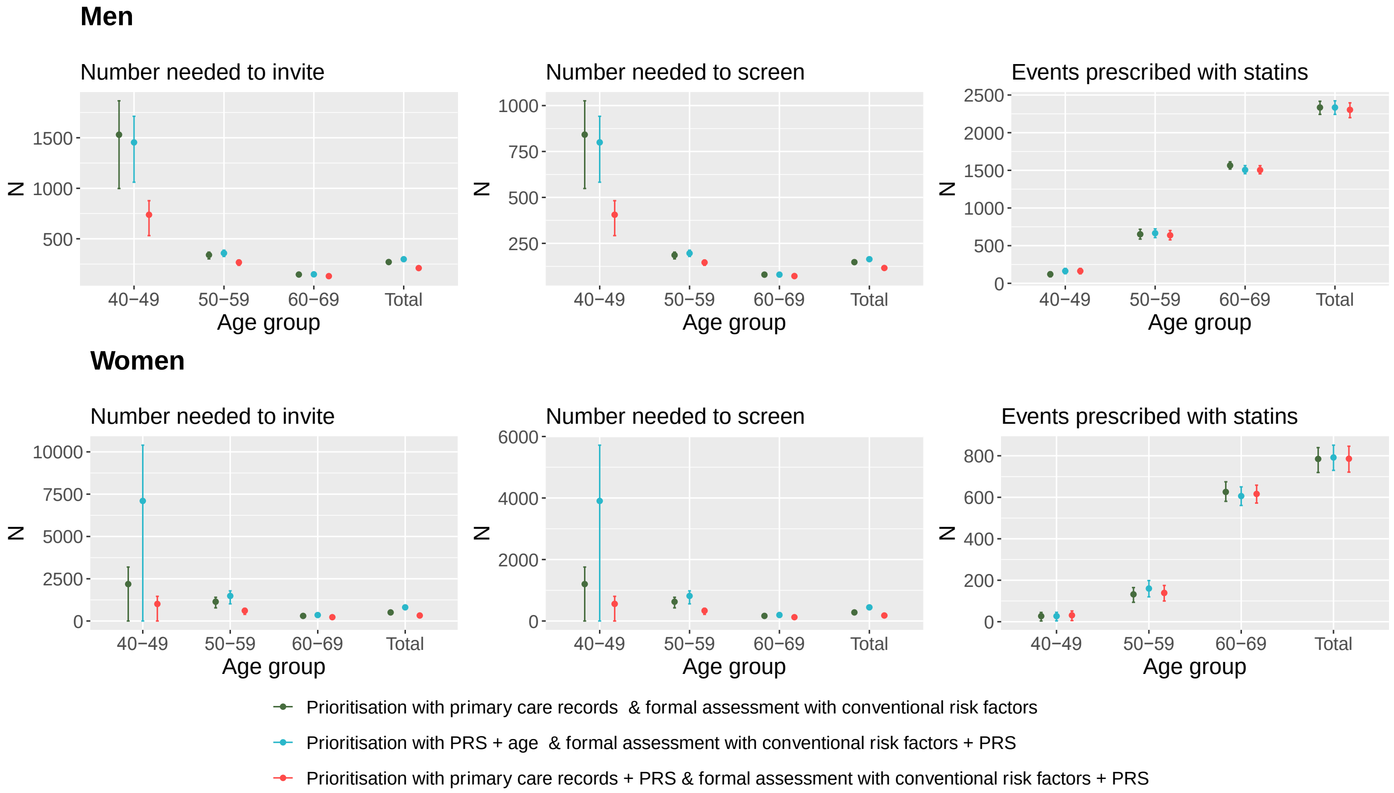

Abbreviations: NNS, number needed to screen; NNI, number needed to invite; PRS, polygenic risk score.

95% confidence intervals are represented by vertical lines. Age group and sex specific prioritisation thresholds were defined as the level such that the expected false negative rate was controlled to be 5%. NNI and NNS assumes 50% statin compliance, and half of all individuals invited for formal assessment attend.

Figure S7. Number needed to invite, number needed to screen and number of events identified after prioritising for a formal CVD assessment, including all individuals without a primary care record for any one of SBP, HDL, total cholesterol or BMI, in a hypothetical population of 100,000 individuals in England.

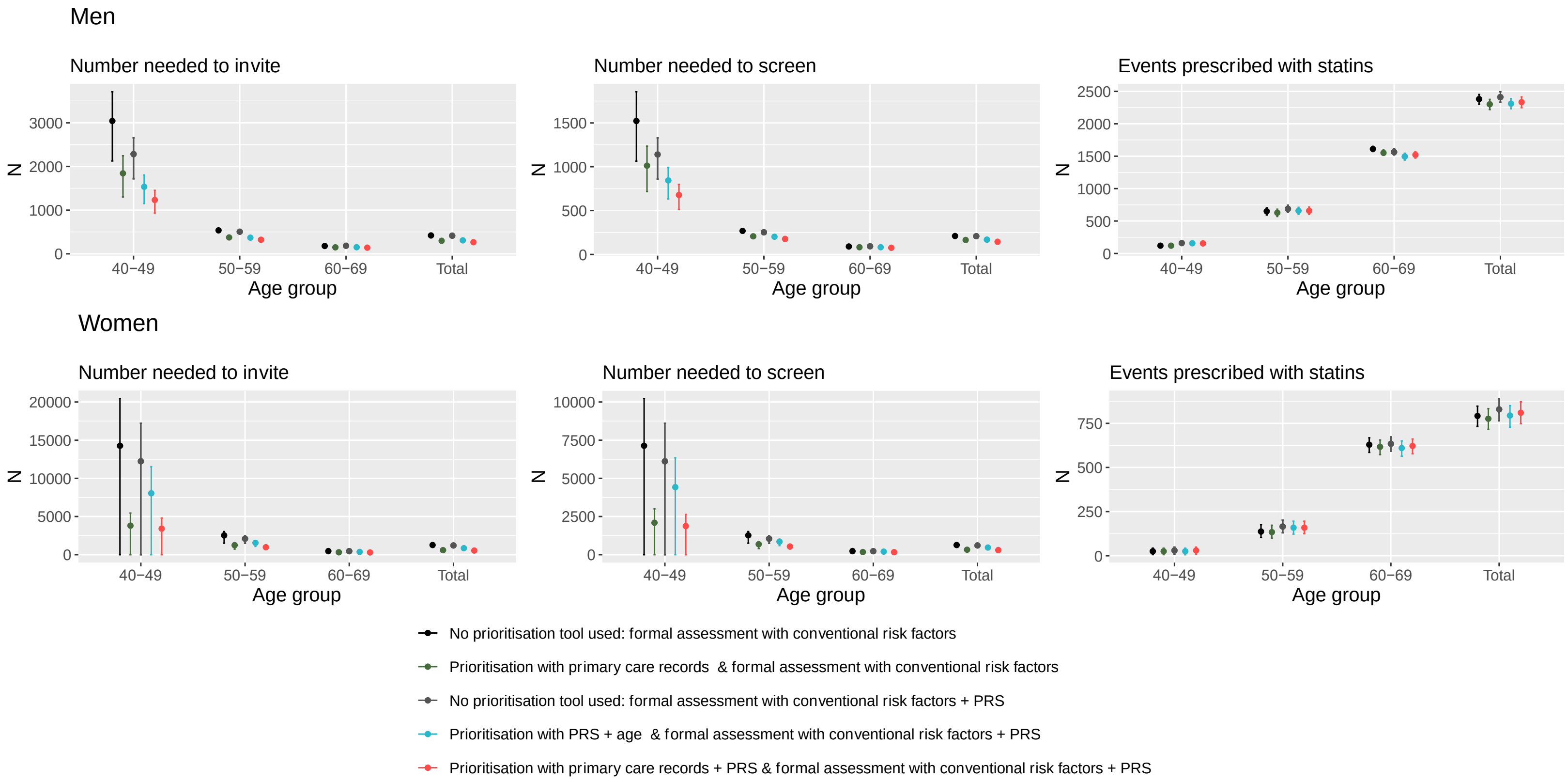

Abbreviations: HDL, high-density lipoprotein; NNI, number needed to invite; NNS, number needed to screen; PRS, polygenic risk score; SBP, systolic blood pressure.

95% confidence intervals are represented by vertical lines. Age group and sex specific prioritisation thresholds were defined as the level such that the expected false negative rate was controlled to be 5%. NNI and NNS assumes 50% statin compliance, and half of all individuals invited for formal assessment attend.
